# Gain of BDNF Function in Engrafted Neural Stem Cells Promotes the Therapeutic Potential for Alzheimer’s Disease

**DOI:** 10.1038/srep27358

**Published:** 2016-06-06

**Authors:** Cheng-Chun Wu, Cheng-Chang Lien, Wen-Hsien Hou, Po-Min Chiang, Kuen-Jer Tsai

**Affiliations:** 1 Institute of Basic Medical Science, College of Medicine, National Cheng Kung University, Tainan, Taiwan; 2 Institute of Clinical Medicine, College of Medicine, National Cheng Kung University, Tainan, Taiwan; 3 Institute of Neuroscience, National Yang-Ming University, Taipei, Taiwan; 4 Center of Clinical Medicine, National Cheng Kung University Hospital, College of Medicine, National Cheng Kung University, Tainan, Taiwan

## Abstract

Stem cell-based therapy is a potential treatment for neurodegenerative diseases, but its application to Alzheimer’s disease (AD) remains limited. Brain-derived neurotrophic factor (BDNF) is critical in the pathogenesis and treatment of AD. Here, we present a novel therapeutic approach for AD treatment using BDNF-overexpressing neural stem cells (BDNF-NSCs). *In vitro*, BDNF overexpression was neuroprotective to beta-amyloid-treated NSCs. *In vivo*, engrafted BDNF-NSCs-derived neurons not only displayed the Ca^2+^-response fluctuations, exhibited electrophysiological properties of mature neurons and integrated into local brain circuits, but recovered the cognitive deficits. Furthermore, BDNF overexpression improved the engrafted cells’ viability, neuronal fate, neurite complexity, maturation of electrical property and the synaptic density. In contrast, knockdown of the BDNF in BDNF-NSCs diminished stem cell-based therapeutic efficacy. Together, our findings indicate BDNF overexpression improves the therapeutic potential of engrafted NSCs for AD via neurogenic effects and neuronal replacement, and further support the feasibility of NSC-based *ex vivo* gene therapy for AD.

Alzheimer’s disease (AD) is a progressive neurodegenerative disease and is a leading cause of dementia in the elderly. It leads to the loss of synapses and neurons, resulting in the impairment of learning and memory^1^. Despite the high prevalence of AD and extensive studies, no curative treatment exists. Recently, stem cell-based therapy has emerged as a promising new therapeutic approach for AD treatment; the transplanted cells, derived from various sources, can ameliorate the cognitive deficits in mouse models of AD by trophic effects, beta-amyloid clearance, or inflammation modulation^2^,^3^,^4^,^5^. However, the engrafted cells’ therapeutic potential for neuronal replacement in the AD brain is still not well defined.

In an AD brain, the accumulations of toxic amyloid-beta (Aβ) protein and various inflammatory mediators may interfere with the survival and neuronal fate of endogenous and engrafted stem cells^6^,^7^,^8^,^9^. Although previous approaches provide therapeutic benefits, a long-term goal for stem cell research in AD should be to achieve neuronal compensation and neural circuitry reconstruction^10^. Thus, the modulation of engrafted cells’ viability, neuronal fate, and electrophysiologically functional maturation may be critical issues in stem cell-based therapy for AD.

Brain-derived neurotrophic factor (BDNF) is a neurotrophin that modulates the survival of stem cells and progenitors, neurogenesis and neuronal differentiation, the branching and survival of differentiated neurons, and the formation and maturation of the dendritic spine and synapses. Thus, BDNF influences learning and memory^11^. Several studies have reported that reduced BDNF levels correlate with the pathogenesis of AD in animal models and patients^12^,^13^. Based on these findings, BDNF has been proposed as a target for developing therapeutic strategies for AD and other neurological disorders^14^,^15^,^16^,^17^,^18^, but its implication in gain of therapeutic potential of engrafted cells for AD is still unknown.

The feasibility of *ex vivo* gene therapy in patients with AD using autologous fibroblasts to deliver nerve growth factor has also been reported^19^. In contrast to fibroblasts, neural stem cells (NSCs) have a greater potential for migration and neural compensation as the vector in *ex vivo* gene therapy; however, the practical application of NSCs for AD remains unexplored^20^,^21^.

Therefore, the present study aims to evaluate whether BDNF overexpression improves the therapeutic potential of NSC-based therapy, and to characterize the role of BDNF in engrafted NSCs. Here, we used a transgenic AD mouse model to assess whether engrafted cells with BDNF overexpression enhance the therapeutic efficacy via the amelioration of AD-related behavioral outcomes. Furthermore, we investigated the effects of BDNF on the engrafted cells’ viability, fates, electrophysiological properties, and the ability to recover the BDNF level and synaptic density. Our results indicated that AD mice transplanted with BDNF-overexpressing NSCs (BDNF-NSCs) exhibited a better recovery in terms of their behavioral outcomes since the engrafted BDNF-NSCs were better able to survive, differentiate into neurons with a higher neurite complexity, and recovered more the synaptic density and BDNF level than control NSCs. These may provide new insights into stem cell-based therapy and *ex vivo* gene therapy.

## Results

### Establishment of BDNF Overexpressing NSCs and *in Vitro* Assays

To investigate the role of BDNF in the therapeutic potential of NSCs-based therapy for AD, we first cultured NSCs/progenitors (collectively called NSCs) and expanded their number from the hippocampi of postnatal day 1 of green fluorescent protein (GFP) expressing transgenic mice (please see Supplementary Fig. S1), and then established BDNF overexpressing NSCs by electroporation of BDNF construct and antibiotic selection. Another bone vector-only construct was also electroporated as a control (Control-NSCs). We confirmed the BDNF overexpression and expressional efficiency (Supplementary Figs S2a–c and S3) on NSCs and found BDNF overexpression improved the neuronal fate in term of neural differentiation (Supplementary Fig. S4). Next, we determined whether BDNF overexpressing existed neuroprotective effect for NSCs in AD brain, Aβ_42_ was added into the culture medium to mimic the microenvironment of AD *in vitro.* The data showed that both kinds of NSCs were damaged in a dose-dependent manner; however, BDNF gene transfer had neuroprotective effects and improved NSC viability, neuronal fate, and neurite outgrowth in response to Aβ toxicity in term of neural differentiation (Supplementary Fig. S5).

### BDNF Overexpression Increases the Therapeutic Potential of Engrafted NSCs by Attenuating Cognitive Deficits

To understand the therapeutic potential of engrafted NSCs with or without BDNF overexpression, we stereotactically transplanted both kinds of NSCs into the hippocampus of 16-month-old Tg2576 mice (referred to as Tg+BDNF-NSCs and Tg+Control-NSCs mice), a transgenic mouse model of AD with a Swedish form of the amyloid precursor protein mutation^22^. Additional age-matched AD mice and non-Tg mice were injected with an equivalent volume of vehicle as controls (referred to as Tg+Vehicle and Non-Tg+Vehicle mice). Before transplantation, the markers of the NSCs were confirmed by immunocytochemistry (ICC) using antibodies against Nestin and Sox2 (Supplementary Fig. S2d,e).

**Figure 1:**
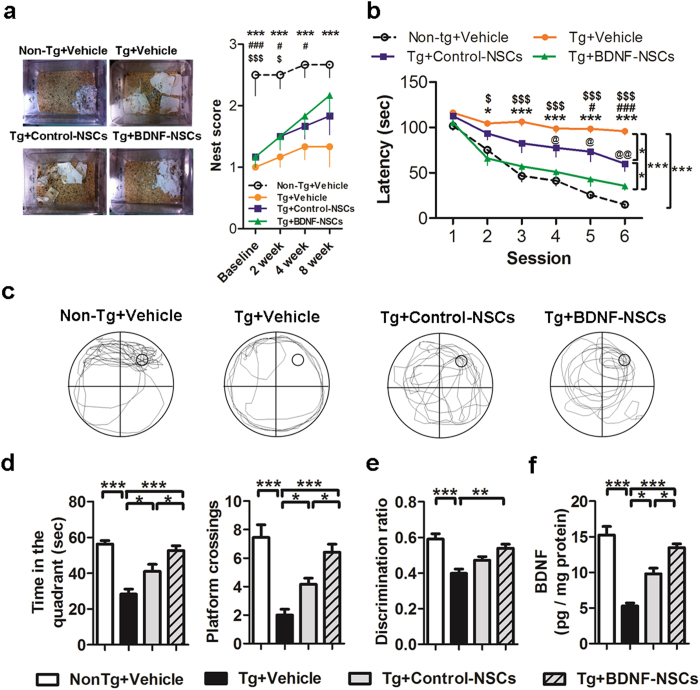
BDNF-NSCs Exhibit More Therapeutic Potential than Control-NSCs to Recover the Cognitive Deficits of AD Mice. (**a**) Representative pictures and scores of the nest construction test (n = 6 per group; *Non-Tg+Vehicle mice vs. Tg+Vehicle mice, ^#^Non-Tg+Vehicle mice vs. Tg+Control-NSCs mice, ^$^Non-Tg+Vehicle mice vs. Tg+BDNF-NSCs mice). (**b**) The escape latency to find the hidden platform in the MWM task (n = 12 per group; *Non-Tg mice vs. Tg+Vehicle mice, ^#^Tg+Vehicle mice vs. Tg+Control-NSCs mice, ^$^Tg+Vehicle mice vs. Tg+BDNF-NSCs mice, ^@^Tg+Control-NSCs mice vs. Tg+BDNF-NSCs mice). The multiple comparisons test confirmed the significant differences between each group. (**c**) Representative data of the search path in the probe trial test. (**d**) The records of the search time in the target quadrant and the number of crosses of the probe location in the probe trial test (n = 12 per group). (**e**) The discrimination ratio of the novel object recognition test (discrimination index = novel object exploration time/total exploration time) (n = 12 per group). (**f**) ELISA for the hippocampal BDNF level (n = 12 per group). All results are presented as the mean ± SEM. * or one symbol represent *p* < 0.05, ** or two symbols represent *p* < 0.01, *** or three symbols represent *p* < 0.001. The data of escape latency and nest construction were analyzed by two-way ANOVAs with Bonferroni post-tests, the others were analyzed by one-way ANOVAs with Tukey’s post hoc tests.

To investigate the effects of BDNF on the therapeutic potential of engrafted NSCs, we first observed the nest construction behavior in AD mice at 2, 4, and 8 weeks after NSC transplantation. The data showed that AD mice failed to exhibit nesting behavior at each time point after transplantation, which was parallel to the finding of a previous study^23^. Transplanting either kind of NSC improved this deficit in AD mice, but Tg+BDNF-NSCs mice revealed an 18% greater improvement than Tg+Control-NSCs mice (Fig. 1a).

Next, we evaluated whether engrafted NSCs ameliorate the spatial memory deficit in AD mice by using the Morris water maze (MWM) task at 8 weeks after NSC transplantation. Within six training sessions, the quantified escape latency indicated that Tg+Vehicle mice had difficulties in finding the platform compared to Non-Tg+Vehicle mice. Although both kinds of transplanted NSCs rescued the spatial memory deficits in AD mice, Tg+BDNF-NSCs mice exhibited shorter escape latency compared with Tg+Control-NSCs mice (Fig. 1b). Furthermore, we assessed the memory consolidation of AD mice at 24 h after escape training using the probe trial test. The video-based tracing paths indicated that Tg+Vehicle mice failed to acquire the platform location compared to Non-Tg+Vehicle mice, and that mice with either kind of transplanted NSC revealed improved memory for the platform location (Fig. 1c). Notably, Tg+BDNF-NSCs mice had longer search times in the target quadrant and had more frequent escape paths that crossed the platform location than Tg+Control-NSCs mice (Fig. 1d), suggesting that BDNF-NSC transplantation may have more therapeutic potential for improving the spatial memory and memory consolidation deficits associated with AD. Additionally, in the novel object recognition (NOR) test, only BDNF-NSCs transplantation revealed any therapeutic potential in this declarative memory test (Fig. 1e).

We also found that Tg+Vehicle mice exhibited significantly reduced BDNF levels compared to Non-Tg+Vehicle mice. Whereas both kinds of transplanted NSCs recovered the hippocampal BDNF level, Tg+BDNF-NSCs mice showed higher levels than Tg+Control-NSCs mice (Fig. 1f). Remarkably, the hippocampal BDNF level correlated with the cognitive functional recoveries in the escape latency and probe trial tests (Table S1). These results provided evidence that BDNF overexpression improves the therapeutic potential of engrafted NSCs, and that BDNF is essential for NSC-based therapy.

Moreover, to further characterize the effects of BDNF in engrafted cell-mediated cognitive recovery, we investigated the long-term therapeutic efficacy of both types of transplantations at 3 months after engraftment. The data showed that engrafted cells with BDNF overexpression were still able to ameliorate the cognitive deficits of AD mice in the MWM test (Supplementary Fig. S6a,b) and the NOR test (Supplementary Fig. S6c), but the engrafted control cells lost their therapeutic potential, suggesting the importance of BDNF for achieving the long-term therapeutic goal.

### Engrafted NSCs Survive and Migrate in the Hippocampus of AD mice

**Figure 2:**
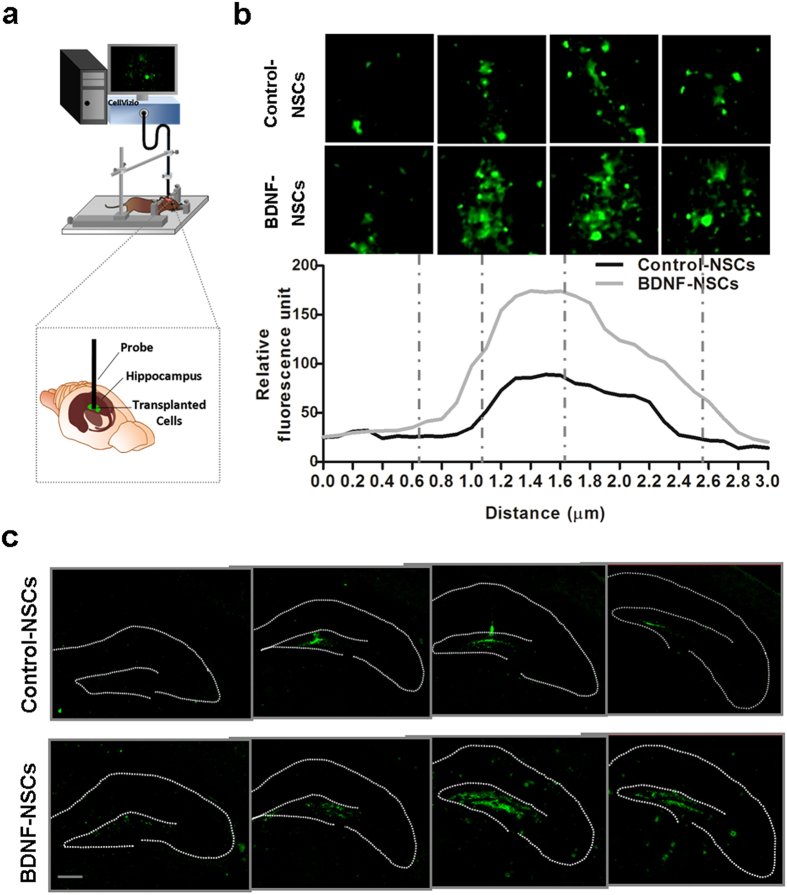
Engrafted NSCs Survive and Migrate in the AD Hippocampus. (**a**) Schematic drawing represents how to use the fluorescence endoscopic confocal microscopy system on living AD mice. (**b**) Top, representative images of living engrafted GFP-expressing cell distributed from top to bottom in the hippocampus of Tg+Control-NSCs and Tg+BDNF-NSCs mice at 4 weeks after transplantation. Each dashed line indicates the vertical extensive position of the acquired view. Quantification of the relative fluorescence units within the vertical extensive path shows that more GFP signals are detected in Tg+BDNF-NSCs mice. (**c**) IF staining of engrafted GFP-expressing cells 4 weeks after transplantation shows that engrafted GFP-expressing cells are able to survive and migrate in the hippocampus of AD mice. Scale bar: 200 μm.

To understand how engrafted NSCs improve the cognitive deficits of AD mice, we investigated the survival, movement, and morphology of engrafted NSCs in the hippocampus of living AD mice using a fluorescence confocal endoscope at 4 weeks after transplantation. We delivered the endoscope probe into the hippocampus of anesthetized mice along the previously transplanted path at a consistent speed (0.1 μm/s) (Fig. 2a), and the series vertical view data showed numerous GFP-expressing cells within the hippocampus (Fig. 2b; see Supplementary Video S1). Upon comparing the number and distribution of surviving GFP-expressing cells in both kinds of NSC-transplanted mice, we observed that the BDNF-NSCs exhibited an increased number of cells with a more widespread distribution in the hippocampus of AD mice. Quantification of data using relative fluorescence units showed that the level of GFP units was higher in the hippocampus of Tg+BDNF-NSCs mice (Fig. 2b), implying that BDNF gene transfer improved the movement and survival of the engrafted NSCs. Furthermore, we investigated the distribution of engrafted NSCs in the coronal view by immunofluorescence staining (IF staining) at 4 weeks after transplantation. The data showed that most of the engrafted NSCs were distributed in the dentate gyrus (DG) within the dorsal to ventral hippocampus (Fig. 2c), with the engrafted BDNF-NSCs displaying a higher number of surviving cells and more extensive migration than engrafted Control-NSCs.

### BDNF Overexpression Improves the Viability and Neuronal Fate of Engrafted NSCs

**Figure 3:**
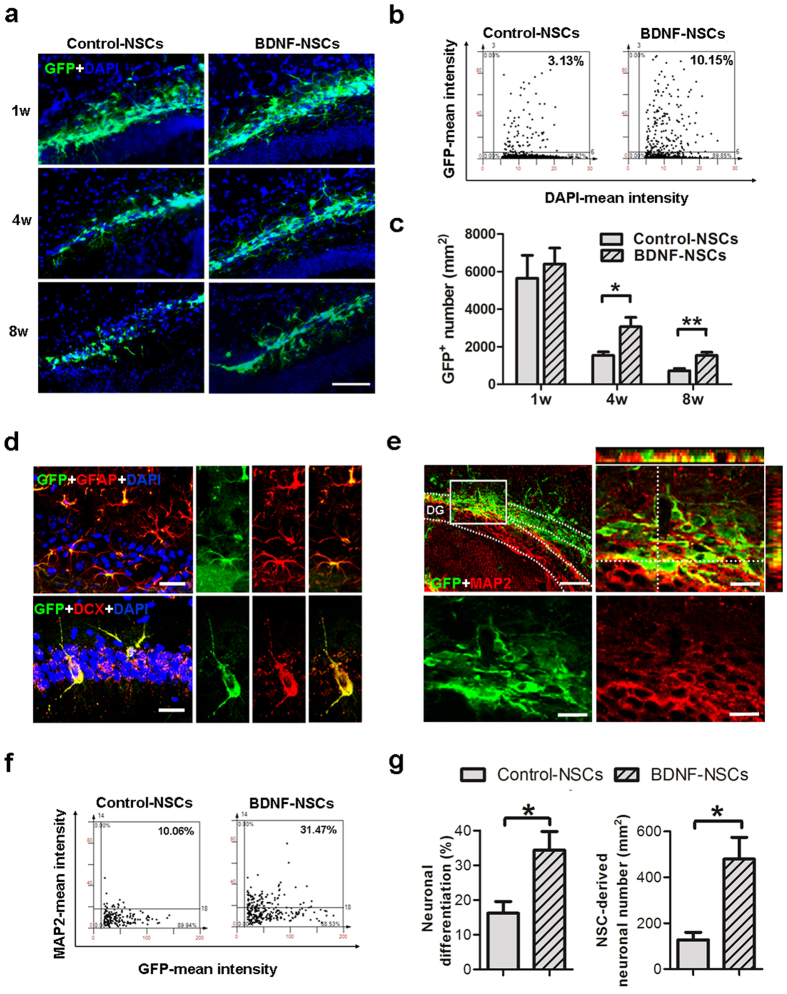
BDNF Overexpression Improves the Viability and Neuronal Fate of Engrafted NSCs. (**a**) IF staining for engrafted GFP-expressing cells in the DG of AD mice at 1, 4, and 8 weeks after transplantation. Scale bar: 200 μm. (**b**) Representative dot plot of IF staining for GFP and DAPI from a flow cytometry-like analysis system (TissueFAXS and TissueQuest) at 8 weeks after NSC transplantation. (**c**) Accumulative mean GFP-expressing cell number at 1, 4, and 8 weeks after NSC transplantation (n = 6 per group at each time point; results represent the mean ± SEM; *p < 0.05 **p < 0.01 by unpaired Student’s *t*-tests). (**d**) IF staining for confocal microscopy indicates that engrafted GFP-expressing cells display DCX and GFAP at 4 weeks after transplantation, confirming their ability to differentiate into neural lineages. Scale bar: 20 μm. (**e**) IF staining of GFP and MAP2 for engrafted cells in the DG of AD mice at 8 weeks after transplantation. The dashed line encloses the granule cell layer (upper-left panel, bar: 100 μm). The blocked area is presented as an amplified orthogonal view (upper-right panel, bar: 20 μm). The immunoreactivities of GFP and MAP2 are presented in separate lower panels (Bar, 20 μm). (**f**) Representative dot plot of the IF staining for GFP and MAP2 at 8 weeks after NSC transplantation. (**g**) Accumulative mean ratio and number of GFP and MAP2 double-positive cells (n = 6 per group; results represent the mean ± SEM; *p < 0.05 by unpaired Student’s *t*-tests).

To characterize the role of BDNF in the survival of the engrafted NSCs *in vivo*, we checked the number of surviving GFP-expressing cells at 1, 4, and 8 weeks after transplantation using IF staining. Remarkably, we conducted a flow cytometry-like system to acquire (TissueFAXS) and analyze (TissueQuest) the IF staining data of hippocampal sections in order to accurately quantify the surviving cells by collecting each positive signal within interested regions on each specimen (Fig. 3a,b)^24^. The results indicated that the engrafted cell viability decreased over time in the hippocampus of AD mice. Of these, BDNF overexpression appeared to be neuroprotective for the engrafted NSCs, as the number of surviving BDNF-overexpressing cells was higher than the number of control cells at 4 and 8 weeks after transplantation (Fig. 3c).

Next, we investigated the potential of engrafted NSCs to differentiate into functional neurons. We found that both kinds of engrafted NSCs were able to differentiate into astrocytes and neuroblasts at 4 weeks after transplantation and were immunoreactive to GFAP and DCX (Fig. 3d). Further, to understand the effects of BDNF on the neuronal fate of engrafted NSCs, we determined the neuronal differentiation ratio in both kinds of NSCs at 8 weeks after transplantation by IF staining for MAP2 and GFP. The parts of the engrafted cells that were immunoreactive to MAP2 were widely distributed in the DG and morphologically integrated into the granule cell layer (GCL) of the AD hippocampus (Fig. 3e). The flow cytometry-like based quantified data indicated that BDNF gene transfer improved the neuronal fate of engrafted NSCs in the AD hippocampus, with BDNF-NSCs displaying a higher ratio of neuronal differentiation and deriving more neurons in the AD brain compared to Control-NSCs (Fig. 3f,g). Notably, we tried to identify whether engrafted NSCs could help Aβ clearance, but the data did not support this opinion (Supplementary Fig. S7).

To further clarify the effects of engrafted NSCs’ survival and fate in the therapeutic potential of cell therapy, we analyzed the correlations between the cognitive functional recoveries and engrafted NSCs. This result showed that the number of surviving NSCs and the number and ratio of neuronally differentiated cells correlated with the cognitive functional recoveries in the escape latency and probe trial tests (Supplementary Table S1). These findings highlight that viability and neuronal fate of engrafted cells are essential for the feasibility of NSC-based therapy, and suggest that BDNF is crucial for improving the therapeutic potential of engrafted NSCs.

### BDNF Overexpression Improves the Engrafted NSC-mediated Synaptic Density Recovery

**Figure 4:**
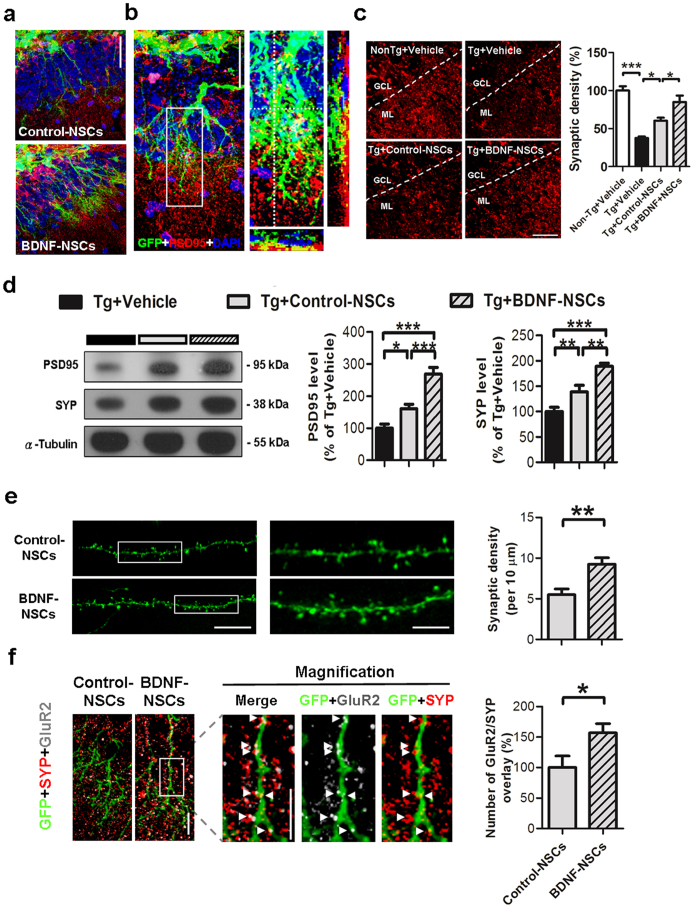
Engrafted NSC-derived Neurons Form Synapses and Ameliorate the Synaptic Loss in the Hippocampus of AD Mice. (**a**) Representative figure of engrafted Control-NSC- and BDNF-NSC-derived neurons integrated into the granule cell layer. Scale bar: 50 μm. (**b**) Left, confocal microscopy z-stack analysis-based IF staining of GFP and PSD95 at 8 weeks after NSC transplantation. Scale bar: 50 μm. Right, the enclosed area is presented as an amplified view with orthogonal projection. (**c**) Left, IF staining of PSD95 in the hippocampal molecular layer at 8 weeks after NSC transplantation. Scale bar: 25 μm. Right, the synaptic density of each view was accumulated and quantified (n = 6 per group; results presented as the mean % ± SEM). (**d**) Representative western blot data and quantification results for the hippocampal level of PSD95 and SYP in AD mice (n = 6 per group; results presented as the mean % ± SEM). (**e**) Representative image of synaptic spines from Control-NSC- and BDNF-NSC-derived neurons. Bar, 15 μm. Magnified images at right side present the region of enclosed white square. Bar, 5 μm. Quantification of the synaptic spine density. (Control-NSCs n = 24 from 3 mice, BDNF-NSC n = 27 from 3 mice) (**f**) Representative image of IF staining for GluR2 and SYP on the two types of engrafted cell. Magnified images present the region of enclosed white square in the respective immunoreactivity from BDNF-NSC. Arrowheads point to the GluR2 and SYP overlays. Bar, 10 μm. (Control-NSCs n = 21 from 3 mice, BDNF-NSC n = 24 from 3 mice; data represented by BDNF-NSC; results presented as the mean % ± SEM). *p < 0.05, **p < 0.01, ***p < 0.001. (**c,d**) Statistical analyses by one-way ANOVAs with Tukey’s post hoc tests. (**e,f**) statistical analyses by unpaired-t test.

Synaptic loss is one of the pathological characteristics of AD and correlates with the degree of dementia in animal models and patients with AD^13^,^25^. In order to know whether both kinds of engrafted cells formed synaptic connections with endogenous brain parenchyma, we performed IF staining of post-synaptic density protein 95 (PSD95) and GFP at 8 weeks after NSC transplantation. In the GCL, both kinds of engrafted cells with matured granule cell morphology integrated into the layer and extended the highly elaborate dendrite structure to the molecular layer (Fig. 4a,b), especial for more evident in BDNF-NSC-derived cells. In addition, the engrafted cells were surrounded by the post-synaptic terminals of endogenous neurons, and part of the PSD95 immunoreactivity was colocalized with the GFP immunoreactivity (Fig. 4b), implying that the engrafted cells were capable of forming synaptic connections with endogenous cells in the hippocampus of AD mice.

Next, to evaluate whether NSC transplantation improved the synaptic density in the hippocampus of AD mice, we investigated the synaptic density in the molecular layer by IF staining of PSD95. The results showed that the Tg+Vehicle mice exhibited reduced synaptic density compared to the Non-Tg+Vehicle mice, and that both kinds of transplanted NSCs improved the recovery of synaptic density, but the Tg+BDNF-NSCs mice at a higher recovery level than Tg+Control-NSCs mice (Fig. 4c). Furthermore, we determined the synaptic protein level of hippocampal lysates by western blot data of synaptophysin (SYP) and PSD95; the data confirmed that BDNF-NSC transplantation recovered more of the hippocampal level of synaptophysin and PSD95 than Control-NSC transplantation did (Fig. 4d). These findings suggest that the BDNF-NSCs are better able to recover the synaptic loss in the hippocampus of AD mice, supporting the idea that BDNF-NSCs exhibit better therapeutic potential in amelioration of the synaptic loss of AD mice.

To further characterize the role of BDNF on engrafted cell-mediated synaptic recovery, we investigated the development of synaptic density on the engrafted cells. The data indicated that both types of engrafted cells could develop the structure of synaptic spines 8 weeks after transplantation, but cells with BDNF overexpression displayed a higher density of synaptic spines (Fig. 4e), suggesting that BDNF improves the synaptic input in engrafted cells. Furthermore, given that AMPA receptors (AMPARs) are highly correlated with excitability during neuronal maturation^26^ and that GluR2 type receptors are the major subunit of AMPAR and are associated with maturation^27^, the amount of GluR2 immunoreactivity on engrafted cells that contact endogenous pre-synaptic immunoreactivity of SYP was analyzed. Comparing both transplantations, engrafted BDNF-NSC-derived neurons exhibited more numbers of GluR2 and SYP overlay than control cells did (Fig. 4f). This data suggests that BDNF overexpression improves the development of excitatory synapses with AMPAR in engrafted cells, implying that BDNF-NSC-derived neurons reestablish more excitatory synapses in the AD hippocampus and display better excitability.

### Engrafted Cells Are Involved in the Ca^2+^-mediated Functional Network of Host

**Figure 5:**
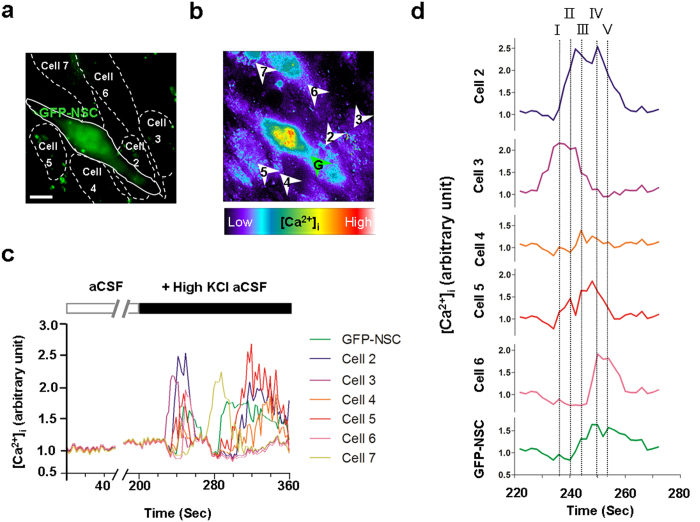
Engrafted Cells Integrate into the Ca^2+^-mediated Functional Network of Host. (**a**) Illustration of the engrafted GFP expressing cells and the endogenous cell borders in the hippocampal DG. Bar, 10 μm. Data represented by BDNF-NSC. (**b**) Image of radiometric calcium orange: brighter color represents higher responses of calcium fluctuations. Arrowheads point to the quantified regions of interest in the measurements of calcium fluctuations. (**c**) [Ca^2+^]_i_ fluctuation responses of engrafted cells (green) and endogenous cells (cell 2–7). High KCl triggered the rise in [Ca^2+^]_i_ and induced temporary related oscillations in the engrafted cell and endogenous cells. (**d**) [Ca^2+^]_i_ traces (220–270 seconds) are magnified in **d**. The chronologically overlapping events from the engrafted cell and neighboring cells are represented by dotted lines I-V.

Given that engrafted cell communication with endogenous cells by Ca^2+^-mediated activity is essential for reestablishment of functional networks in the brain^28^, we used the calcium indicator, calcium orange, to trace the calcium fluctuations between engrafted and endogenous cells for investigating whether the engrafted NSCs are able to integrate functionally into the endogenous neuronal network. Four weeks after transplantation, both kinds of engrafted cells exhibited restricted spontaneous fluctuations of intracellular calcium ([Ca^2+^]_i_) during control conditions of artificial cerebrospinal fluid (aCSF) (Fig. 5a–c, Supplementary Video S2; Control-NSCs n = 21 from 5 mice, BDNF-NSCs n = 24 from 5 mice). However, application of high potassium chloride elicited responses of calcium fluctuation from engrafted and endogenous cells (Fig. 5c and Supplementary Video S2). Images of radiometric calcium orange [Ca^2+^]_i_ indicated the engrafted cells were able to integrate into Ca^2+^-mediated functional networks. The Ca^2+^-waves propagated within engrafted cells and neighboring endogenous cells, and the fluorescent peak analysis also showed temporal overlaps between engrafted cells and endogenous cells (Fig. 5d, Supplementary Video S2). This suggests that the engrafted cells have Ca^2+^-mediated interactions with endogenous cells and implies its integration into the functional network.

### Engrafted Cells Behave as Functional Neurons and BDNF Overexpression Improves Its Excitability and Neurite Complexity

**Figure 6:**
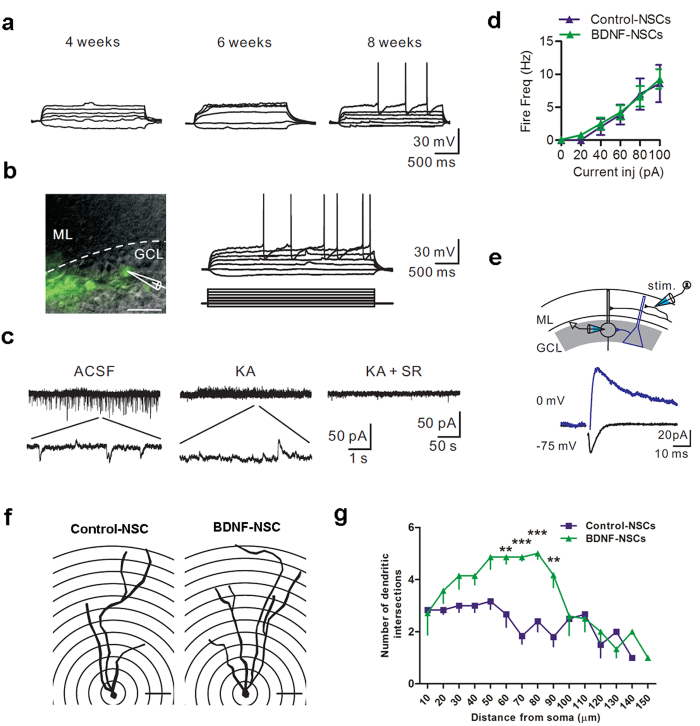
Engrafted NSC-derived Neurons Display the Electrophysiological Properties of Mature Neurons and Exhibit Neurite Complexity. (**a**) Representative voltage responses of engrafted cells at 4, 6, and 8 weeks after transplantation. Data represent by BDNF-NSC. (**b**) Left, a representative recording of an engrafted cell. Scale bar: 20 μm. Right top, voltage responses of the recorded cell; right bottom, 2 s current protocol, 10 pA steps. Data represent by BDNF-NSC. (**c**) Spontaneous synaptic currents recorded from the recorded cell in (**b**) holding at −55 mV. The enlarged trace indicates the spontaneous EPSCs and IPSCs. The spontaneous events were blocked by the addition of AMPA/NMDA (KA) and GABAergic receptor blockers (SR). (**d**) Mean firing frequency of Control-NSC- and BDNF-NSC-derived neurons 8 weeks after transplantation (Control-NSCs, n = 16 from 5 mice; BDNF-NSCs, n = 19 from 7 mice). (**e**) Top, experimental diagram. Stimulation electrode was placed in the molecular layer at a distance of 100 μm from the recorded cell. Bottom, the evoked AMPAR-/NMDAR- (black) or GABAR-mediated (blue) currents of the recorded cell in (**b**) (average from 10 sweeps). (**f**) Representative reconstructed cell of Control-NSC-derived and BDNF-NSC-derived neuron with the given radius. Scale bar: 20 μm. (**g**) Sholl analysis for both kinds of cell-derived neurons. (Control-NSCs, n = 6 from 3 mice; BDNF-NSCs, n = 7 from 3 mice; results presented as the mean ± SEM). Statistical analyses by two-way ANOVAs with Bonferroni post-tests.

To test whether engrafted NSC-derived neurons can integrate into the pre-existing neural circuits of the host brain and display functional activities, we performed whole-cell patch-clamp recordings on acute brain slices obtained from transplanted mice. Initially, we successfully detected action potential (AP) firing and spontaneous postsynaptic currents in both kinds of engrafted NSC-derived neurons in the hippocampal GCLs of non-Tg mice 4 weeks after transplantation (Fig. S8; Control-NSCs n = 5 from 3 mice, BDNF-NSCs n = 5 from 3 mice). However, both kinds of engrafted cells insufficiently generated APs 4 and 6 weeks after transplantation in the DG of AD mouse (Fig. 6a; Control-NSCs n = 5 from 3 mice, BDNF-NSCs n = 5 from 3 mice) and revealed immature granule cell properties (input resistance > 1 GΩ). Thus, we speculated that the AD brain’s microenvironment is more detrimental for the neuronal maturation of engrafted cells than that of non-Tg brains. Up to 8 weeks after NSC transplantation, we detected that 32.5 ± 15.4% of engrafted control cells and 84.3 ± 10.2% of engrafted cells with BDNF overexpression were able to generate APs in response to depolarizing current pulse injections, and we found spontaneous postsynaptic activity and electrical properties of functional neurons (Fig. 6a–c, Table 1; Control-NSCs n = 16 from 5 mice, BDNF-NSCs n = 19 from 7 mice). In a comparison of the electrophysiological properties between both kinds of transplantations, cells with BDNF overexpression exhibited a lower input resistance, a higher capacitance, and a higher ratio to generate APs than control cells. Furthermore, comparing the properties in AP firing, BDNF-NSC-derived neurons exhibited a lower AP threshold and higher AP amplitude (Table 1). This is regardless of the data that showed no difference in the mean firing frequency between both kinds of transplantations (Fig. 6d). These findings suggest that BDNF overexpression improves the functional maturation of engrafted cells in the excitability 8 weeks after transplantation into the AD brain.

To examine the ability of transplanted cells received synaptic inputs, we assessed the spontaneous excitatory postsynaptic currents (EPSCs) and inhibitory postsynaptic currents (IPSCs) of the engrafted cell-derived neurons; both Control-NSC- and BDNF-NSC-derived neurons received excitatory and inhibitory inputs, which were blocked after addition of AMPA/NMDA and GABA_A_ receptor blockers (Fig. 6c; Control-NSCs n = 5 from 3 mice, BDNF-NSCs n = 5 from 3 mice). Notably, we further tested if physiological inputs can target the engrafted cell-derived neurons. By stimulating axons in the inner molecular layer, we recorded evoked glutamatergic and GABAergic currents in all engrafted cell-derived neurons (Fig. 6e). Taken together, the above results indicate that the engrafted cell-derived neurons were able to generate APs and receive inputs projecting to the DG or from local GABAergic neurons, and thus integrate into the glutamatergic and GABAergic circuits in the hippocampus of AD mice.

Moreover, we used Sholl analysis to evaluate the dendritic development and neurite complexity by morphological reconstructions of recorded neurons after biocytin-filled recordings. Although both kinds of NSC-derived neurons were able to display matured granule cell morphology with highly branched dendrites (Fig. 6f; Control-NSCs n = 6 from 3 mice, BDNF-NSCs n = 7 from 3 mice), BDNF-NSC-derived neurons exhibited higher neurite complexity than Control-NSC-derived neurons (Fig. 6g), suggesting that BDNF overexpression improves the neurite extension and neuronal morphological maturation of engrafted cell-derived neurons.

### BDNF Is Essential for BDNF-NSC-mediated Cognitive-deficit Amelioration

**Figure 7:**
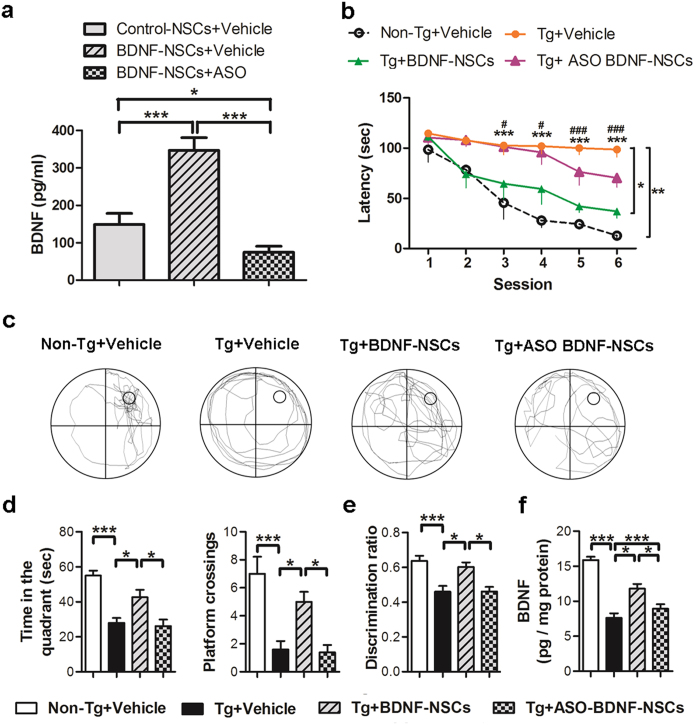
BDNF Is Critical for Engrafted BDNF-NSC-mediated Cognitive Improvements. (**a**) ELISA for the secreted BDNF level following ASO BDNF treatment (n = 5 independent experiments). (**b**) The MWM task-based analysis of the latency for finding the hidden platform (n = 6 per group; *Non-Tg+Vehicle mice vs. Tg+Vehicle mice, ^#^Tg+Vehicle mice vs. Tg+BDNF-NSCs mice). The multiple comparisons test confirmed the significant differences between Tg+Vehicle mice and Non-Tg+Vehicle mice or Tg+BDNF-NSCs mice. (**c**) Representative search path in the probe trial test. (**d**) The records of the search time in the target quadrant and the number of crosses of the probe location in the probe trial test (n = 6 per group). (**e**) The discrimination ratio of the novel object recognition test (n = 6 per group). (**f**) ELISA for the hippocampal BDNF level (n = 6 per group). All results are presented as the mean ± SEM. * or one symbol represent *p* < 0.05, ** or two symbols represent *p* < 0.01, *** or three symbols represent *p* < 0.001. Statistical analyses by one-way ANOVAs with Tukey’s post hoc tests.

To further characterize the critical role of BDNF in the engrafted BDNF-NSC-mediated cognitive improvement of AD mice, we conducted a loss-of-function study using BDNF mRNA anti-sense oligomers to knock down BDNF expression (referred to as ASO BDNF-NSCs) following BDNF overexpression. Before NSC transplantation, we confirmed that the secreted BDNF level in ASO BDNF-NSCs was significantly decreased using ELISAs (Fig. 7a). At 8 weeks after NSC transplantation, the MWM task indicated that ASO BDNF-NSC-transplanted AD mice (Tg+ASO BDNF-NSCs mice) had limited spatial memory recovery in the escape latency (Fig. 7b) and an impaired memory consolidation ability in the probe trial test (Fig. 7c,d) compared to Tg+BDNF-NSCs mice. Furthermore, the novel object recognition test as well as the hippocampal BDNF ELISA indicated that ASO BDNF-NSC transplantation was insufficient to recover the declarative memory and BDNF level of AD mice (Fig. 7e,f), suggest that the engrafted BDNF-NSC-mediated cognitive improvement was blocked by BDNF knockdown.

## Discussion

Our data demonstrate the essential role of BDNF in engrafted NSCs and present a novel approach for improving the therapeutic potential of NSC-based therapy. We found that BDNF gene transfer improved the therapeutic potential of NSCs by increasing graft viability (Figs S5a, 2 and 3a–c), neuronal fate (Figs S4 and 3e–g), and neurite complexity (Figs S5b,c and 6f,g). Further, we showed that BDNF-NSCs transplantation was better able to recover the hippocampal BDNF level (Fig. 1f) and synaptic density (Fig. 4) compared to the transplantation of Control-NSCs. Most importantly, even though both kinds of engrafted NSCs performed electrophysiological properties and integrated into the hippocampal circuit, the electrophysiological properties of BDNF-NSC-derived neurons were better able to behave as functionally matured neurons (Fig. 6, Table 1). Accordingly, BDNF-NSCs were more competent to ameliorate the cognitive deficits (Fig. 1) via a higher potential for neuronal functional replacement and BDNF supplementation. These findings highlight the critical role of BDNF in the therapeutic efficacy of the engrafted cells, and provide insights into the applications of BDNF in stem cell therapy. Compared to a previous NSC-based study in AD reported by Blurton-Jones *et al*.^2^, this study underlines the beneficial effects of BDNF in the fate of engrafted cells for developing as functionally matured neurons that contribute to the AD brain, and it provides further insight into the potential of engrafted cells in acting as functional neurons and integrating into synaptic circuits of the host (Supplementary Table S2).

Multiple lines of evidence indicate that the BDNF level is reduced in the entorhinal cortex and hippocampus of patients with AD^12^,^29^, and show that this reduction correlates with the patients’ scores on the mini-mental state examination^30^. Moreover, since the reduced BDNF level is associated with the low proliferation and differentiation capacities of adult hippocampal stem cells^31^, BDNF recovery is being developed as a strategy for treating AD. Data from the present study are consistent with previous findings, indicating a reduced hippocampal BDNF level in AD mice, as well as a significant correlation between the BDNF level and the recovery of cognitive deficits (Supplementary Table S1), which further support the use of BDNF treatment as a feasible therapeutic approach in AD. Additionally, we showed that engrafted NSCs can differentiate into astrocytes, which are known to modulate synaptic plasticity^32^, excitatory synaptic transmission^33^, and BDNF supplementation^34^. Thus, we speculate that engrafted astrocytes also participate in BDNF supplementation and synaptic plasticity modulation in the hippocampus in AD.

Accumulating evidence suggests that neurocognitive impairments parallel neurogenic deficits in neurological disorders^35^,^36^, and that the microenvironment in the AD brain impedes the survival and neuronal fate of intrinsic or extrinsic stem cells [8]; manipulating the neurogenic potential of stem cells may be a worthwhile strategy in stem cell research for treating neurological diseases^37^. Here, we confirmed that the microenvironment in the AD brain is harmful to the viability and neuronal fate of NSCs (Fig. 3a–c), and cells showed delayed development of electrophysiological properties (Fig. 6) compared to a previous study^38^. However, data from the BDNF-overexpressing cells clearly show the importance of BDNF for the survival, neuronal fate, and functional maturation of the engrafted cells, and further clarify the association between the neurogenic fate of the engrafted cells and the cognitive amelioration in AD mice (Supplementary Table S1). Additionally, by BDNF knockdown, we found that BDNF is indispensable for the therapeutic efficacy of the engrafted cells (Fig. 7). These findings provide evidence of the positive effects of BDNF on the survival and neuronal fate of engrafted NSCs, and further support the neurogenic manipulation approach for increasing therapeutic efficacy.

In the present investigation, using the patch-clamp technique and IF staining, we found that both kinds of engrafted cell-derived neurons gradually differentiated into functional neurons and integrated into the hippocampal circuit after transplantation (Fig. 6; Table 1). We further identified engrafted cell-derived neurons with repetitive, overshooting, low-threshold, short-duration APs, which indicated that the engrafted cells had developed characteristics of mature neuronal excitability, especially for cells with BDNF overexpression^39^. Additionally, we detected GABAergic and glutamatergic spontaneous postsynaptic currents and evoked currents in the engrafted cell-derived neurons (Fig. 6c,e), another significant property of mature functional neurons^40^. This indicates that engrafted cell-derived neurons were integrated into a synaptic network of endogenous neurons. This finding demonstrates the feasibility of neuronal replacement. Furthermore, the input resistance, capacitance, AP firing, and AP threshold are critical characteristics of the process of neuronal development, and they are highly associated with the neuronal maturation status^38^,^41^. In this study, our data showed that BDNF-NSC-derived neurons exhibited more of the above features of neuronal maturation than did control-NSC-derived neurons. Thus, this demonstrates that BDNF overexpression improves the electrophysiological functional maturation of engrafted cells.

Neuronal loss is the major cause of cognitive deficits in AD; thus, the goal of neuronal replacement should be accomplished in stem cell research. Recently, the main cell types being used and studied for AD treatments are mesenchymal stem cells and NSCs, but the goal of replacing the injured neurons in the AD brain has not yet been achieved. The benefits of engrafted mesenchymal stem cells include Aβ clearance and inflammation modulation^3^,^5^, while the benefits of engrafted NSCs are mainly due to BDNF supplementation^42^, even though they are potentially able to differentiate into neural cells. Here, our data showed that the engrafted NSCs were not only able to differentiate into neurons and also integrate into the dentate GCL, display the electrophysiological properties of mature neurons, and form synaptic connections in the hippocampal circuits, suggesting that engrafted neurons may partially replace lost neuronal functions and reconstitute the synaptic circuit, thereby recovering the cognitive deficits.

Synaptic loss is another pathologic hallmark of the AD brain that is highly correlated with the pathogenesis of the disease^43^,^44^. Since BDNF regulates neurite outgrowth and synaptic plasticity, reduction of the BDNF level is also involved in this pathophysiology^45^. However, synaptic dysfunction and synapse loss, unlike neuronal loss, are reversible, highly dynamic, and plastic^18^,^46^,^47^. Our data presented herein reinforce this concept that synaptic density recovery is associated with the BDNF level and cognitive amelioration, providing evidence that NSC-derived neurons can form synaptic connections within the AD brain and recover the synaptic loss by neuronal compensation and BDNF supplementation.

In light of *ex vivo* gene therapy in neurological disorders, this study supports the strategic feasibility of NSC-based *ex vivo* gene therapy for treating neurological disorders because of the neural fate and high migration abilities of NSCs. Therefore, engrafted NSCs can potentially contribute to neuronal compensation and serve as a sufficient vector for delivering BDNF or other therapeutic factors^48^. With regard to the clinical applications, the process of generation of autologous NSCs will be an important issue. Recently, several important studies have reported that autologous NSCs can be acquired from induced pluripotent stem cells^49^,^50^,^51^, human nasal stem cells^52^,^53^, or genetically modified bone marrow-derived mesenchymal stem cells^54^. These findings provide hope for using autologous NSCs combined with gene engineering to treat neurological disorders. However, their therapeutic potential and suitable gene modified targets in clinical applications warrant further studies.

In conclusion, our findings demonstrate that BDNF is essential for the survival, neuronal fate, and morphological and functional maturation of engrafted NSC-derived neurons, and that BDNF gene transfer is a viable approach for improving the therapeutic potential of stem cell-based therapy. Our data support that NSCs are a feasible vector for the development of *ex vivo* gene therapy.

## Materials and Metheds

### AD Mouse Model and Behavioral Tests

Tg2576 mice were purchased from Taconic Company and were bred at the laboratory animal center of National Cheng Kung University (NCKU). Experimental procedures for handling the mice were in accordance with the guidelines of the Institutional Animal Care and Use Committee (IACUC) of NCKU. All experimental protocols were approved by IACUC of NCKU, approved number 103–119. The mice were housed in a room maintained on a 12-h light-dark cycle and fed in *ad libitum*. Sixteen-month-old Tg2576 mice were used in all experiments and age-matched Tg^−/−^ (non-Tg) mice served as the control. For the procedures of behavioral tests, please see supplementary information.

### NSCs Primary Culture, Construction, and Gene Engineering

GFP-expressing NSCs or neural progenitors were derived from a transgenic mouse line with “enhanced” GFP cDNA from The Jackson Labs (Bar Harbor, ME). The GFP-expressing NSCs were harvested from the hippocampi of postnatal day 1 pups and expanded in an adherent type, as described previously^55^. Each batch of NSCs was cultured for 10 days *in vitro*, and then conducted to BDNF or control vector electroporation. To generate the BDNF construct, a human *BDNF* coding region sequence was cloned into the pGEM^®^-T Easy Vector (Promega A1360). Next, the BDNF fragment containing *Xba*I and *Eco*RI sites was cloned from the pGEM^®^-T Easy Vector and ligated into the vector pFUGW (pFUGW_BDNF), which is driven by a human polyubiquitin-C promoter. The pFUGW_BDNF constructs were electroporated into NSCs via the Neon^®^ Transfection System from Thermo (Waltham, MA) and were selected by G418 from Thermo (Waltham, MA) to establish BDNF-NSCs. Another bone vector-only construct was used for generating Control-NSCs. For further details of construction, please see supplementary information.

### NSC Transplantation

The NSC transplantation surgery and stereotactic transplantation followed the methods used in the previous study^2^. Briefly, the cells were trypsinized, washed, titrated, and filtered through a 70 μm mesh before transplantation. The NSCs were then counted and resuspended at 50,000 cells/μL in Hank’s Balanced Salt Solution from Thermo (Waltham, MA). Subsequently, 2 μL of NSCs or vehicle were injected into the hippocampus using a micro-injector attached to a stereotactic apparatus; the transplant coordinates were relative to bregma (anterior/posterior, −2.0; medial/lateral, ±1.80; dorsal/ventral, −2.0 mm).

### Endoscopic Confocal Microscopy

To assess the distribution of engrafted GFP-expressing cells within the hippocampus in living AD mice, endoscopic confocal fluorescence microscopy from Mauna Kea Technologies (Suwanee, GA) was conducted. A flexible probe was attached to a stereotactic apparatus and lowered slowly into the hippocampus along the original path of cell transplantation at a consistent speed. Video data were recorded by CellVizio 488 (Mauna Kea Technologies) and analyzed by ImageCell software (Mauna Kea Technologies) to obtain the relative fluorescence units from each region of interest. The methods for quantification of the relative florescence units are as previously described^56^.

### Western Blotting, ELISA, and IF Staining

To analyze the hippocampal protein level, extracts were prepared from the hippocampus of non-Tg or AD mice. The detailed protocol for western blotting has been described previously^57^. The extracts were analyzed by 8–12% sodium dodecyl sulfate polyacrylamide gel electrophoresis, followed by blot hybridization with the anti-bodies. Image J software was used to quantify the relative intensities of the bands, which were normalized to the intensity of tubulin. The BDNF ELISA was performed following the manufacturer’s protocol from Promega (Madison, WI). To prepare the tissue for IF staining, adult mice were anesthetized and perfused transcardially by PBS and 4% paraformaldehyde. The brain of each mouse was removed and immersed in a 4% PFA solution for 2 h and dehydrated. After preparing the cryosections, the tissues were incubated with a series of primary antibodies. For further details of antibodies, procedures, and data quantification, please see supplementary information.

### Slice Preparation and Electrophysiological Recording

Mice were killed by rapid decapitation in accordance with the guidelines of animal center of National Yang-Ming University. Their brains were rapidly removed, and 300 μm-thick coronal hippocampal slices were cut in ice-cold sucrose solution containing the following (in mM): 87 NaCl, 25 NaHCO_3_, 1.25 NaH_2_PO_4_, 2.5 KCl, 10 glucose, 75 sucrose, 0.5 CaCl_2_, and 7 MgCl_2_ using a vibratome from Dosaka (Kyoto, Japan). Slices were incubated in the sucrose solution (equilibrated with 95% O_2_ and 5% CO_2_) in a holding chamber at 34 °C for 30 min and kept in the same chamber at room temperature (23 ± 2 °C) until used. During the experiments, slices were placed in a recording chamber and superfused with oxygenated aCSF containing the following (in mM): 125 NaCl, 25 NaHCO_3_, 1.25 NaH_2_PO_4_, 2.5 KCl, 25 glucose, 2 CaCl_2_, and 1 MgCl_2_. The recording temperature was 23 ± 2 °C. Recording electrodes (3–7 MΩ) were pulled from borosilicate glass (Harvard Apparatus, Holliston, MA), outer diameter, 1.5 mm; thickness, 0.32 mm. GFP^+^ cells were first identified by the fluorescence with the soma located in the inner molecular layer, granule cell layer, or the hilus under an infrared and differential interference contrast microscope of Olympus BX51WI (Center Valley, PA) coupled with an infrared-sensitive CCD camera (Hamamatsu C7500–50, Shizuoka, Japan). Recorded cells (held at −70 mV in current clamp) were depolarized to generate APs by somatic current pulse injection.

In the experiments where spontaneous EPSCs and IPSCs were recorded, cells were held at −55 mV. When recording evoked glutamatergic/GABAergic currents, a stimulation electrode was placed in the inner molecular layer. Single short (0.1 ms duration) current pulses were delivered to evoke synaptic responses. Cells were held at −75 or 0 mV. Whole-cell patch-clamp recordings were made using a Multiclamp 700B amplifier from Molecular Devices (Sunnyvale, CA). Pipette capacitances of both electrodes were carefully compensated (by ~95%), and series resistance (Rs) was compensated using the automatic bridge balance (readouts after compensation were 9–21 MΩ). Signals were filtered at 4 kHz using the 4-pole low-pass Bessel filter. A Digidata 1440A (Molecular Devices) connected to a personal computer was used for stimulus generation and data acquisition. The sampling frequency was 10 kHz. Pulse sequences were generated by pClamp 10.2 (Molecular Devices). For further details of electrophysiological recording, please see supplementary information.

### Calcium Imaging

The experimental procedure and measurements are similar to that used in previous studies^28^,^58^. Briefly, 4 weeks after control- or BDNF-NSCs transplantation, 150-μm-thick acute brain slices were prepared and incubated with 2 μM calcium orange-AM (Thermo) and 0.02% Pluronic F-127 (Thermo) for 1 h in aCSF and washed for 30 min at 37 °C with oxygen supplementation. During calcium imaging experiments, the temperature was maintained at 37 °C with sustained oxygen supplementation using a specialized chamber (GE Healthcare, Buckinghamshire, UK), and a DeltaVision Elite image system (GE Healthcare) equipped with an inverted microscope (Olympus IX71) and a 40× A-UPO oil-immersion objective (Olympus). Calcium orange indicator was excited at 542 nm and was emitted at 597 nm. The engrafted NSCs were identified by 475 nm excitation (GFP). In the collection of calcium fluctuations, during the first 200 seconds, the spontaneous [Ca^2+^]_i_ oscillations were collected with no additional chemo transmitters or inducers, and the responses of calcium fluctuations were measured after treatment with 90 mM potassium chloride (Sigma). The collected images were stacked and transferred to grey scale or ratio-metric images using MetaMorph image analysis software (Moleculardevises), and the quantification of calcium fluctuations was measured with ImageJ software using the tool that sets the region of interest and multiple measurements.

### BDNF Knockdown by ASOs

BDNF knockdown was performed by the LNA^TM^ longRNA GapmeR from Exiqon (Woburn, MA) that was produced using a custom design. The product sequence of the oligonucleotide was 5′-GCAGCATCTAGGTAAT-3′. The oligonucleotide was eletroporated into cultured NSCs in the working concentration (1 μM) at 48 h after BDNF gene electroporation. The knockdown efficiency was confirmed by BDNF ELISA.

### Statistical Analysis

All data were normally distributed except for whole-cell recording data. All data are shown as the mean ± SEM. In the case of single mean comparisons, data were analyzed by Student’s *t*-tests. For multiple mean comparisons, data were analyzed by one-way ANOVAs with Tukey’s post hoc tests, or by two-way repeated measure ANOVAs with Bonferroni multiple comparison tests. Whole-cell recording data were analyzed by Mann-Whitney tests. All statistical analyses were performed using Prism 4.0 (GraphPad). The statistical significance was set at *p* < 0.05, * or one symbol represents *p* < 0.05, ** or two symbols represent *p* < 0.01, *** or three symbols represent *p* < 0.001.

## Additional Information

**How to cite this article**: Wu, C.-C. *et al*. Gain of BDNF Function in Engrafted Neural Stem Cells Promotes the Therapeutic Potential for Alzheimer’s Disease. *Sci. Rep.* **6**, 27358; doi: 10.1038/srep27358 (2016).

## Supplementary Material

Supplementary Information

Supplementary Dataset 1

Supplementary Dataset 2

## Figures and Tables

**Table 1:** Electrophysiological Properties of Transplanted Control-NSC- and BDNF-NSC-derived Neurons Generating One or More APs Upon Current Injection.

n	Control-NSCs	BDNF-NSCs	*p*value
	16 (from 5 mice)	19 (from 7 mice)	
V_rest_ (mV)	−74.7 ± 1.6	−73.4 ± 1.0	0.45
R_input_ (MΩ)	1388 ± 326	739 ± 205	0.04
C (pF)	20.1 ± 4.1	39.9 ± 6.5	0.03
% of cell with APs	32.5 ± 15.4%	84.3 ± 10.2%	0.02
AP threshold (mV)$	−20.3 ± 1.4	−38.8 ± 2.4	0.001
AP amplitude (mV)$	56.4 ± 4.1	86.0 ± 8.0	0.03
AP half-width (ms)$	1.8 ± 0.1	1.6 ± 0.1	0.60
AHP amplitude (mV)$	8.7 ± 1.3	15.4 ± 1.2	0.01

V_rest_ = resting membrane potential; R_input_ = input resistance; C = capacitance. AP = action potential; AHP = afterhyperpolarization. Statistical analyses by the Mann-Whitney test. ^$^Analysis based on the first AP induced by current injection.
