# Supplementary material for: Gain of BDNF Function in Engrafted Neural Stem Cells Promotes the Therapeutic Potential for Alzheimer’s Disease
Source: Sci Rep. 2016 Jun 6;6:27358. doi: 10.1038/srep27358 (PMC4893631; doi:10.1038/srep27358)
Supplement: Supplementary Information [file srep27358-s1.doc]

**Supplementary Information**

**Gain of BDNF Function in Engrafted Neural Stem Cells Promotes the Therapeutic Potential for Alzheimer’s Disease**

Cheng-Chun Wu^a,b^, Cheng-Chang Lien^c^, Wen-Hsien Hou^c,1^, Po-Min Chiang^a,b,1^,

and Kuen-Jer Tsai^a,b,d^

^a^Institute of Basic Medical Science, College of Medicine, National Cheng Kung University, Tainan, Taiwan

^b^Institute of Clinical Medicine, College of Medicine, National Cheng Kung University, Tainan, Taiwan

^c^Institute of Neuroscience, National Yang-Ming University, Taipei, Taiwan

^d^Center of Clinical Medicine, National Cheng Kung University Hospital, College of Medicine, National Cheng Kung University, Tainan, Taiwan

^1^The authors contributed equally to the study

**Correspondence to:** Kuen-Jer Tsai, Ph.D.

Institute of Clinical Medicine

College of Medicine

National Cheng Kung University

Tainan, Taiwan

Tel: +886-6-2353535-4254

Fax: +886-6-2758781

Email: [kjtsai@mail.ncku.edu.tw](mailto:kjtsai@mail.ncku.edu.tw)


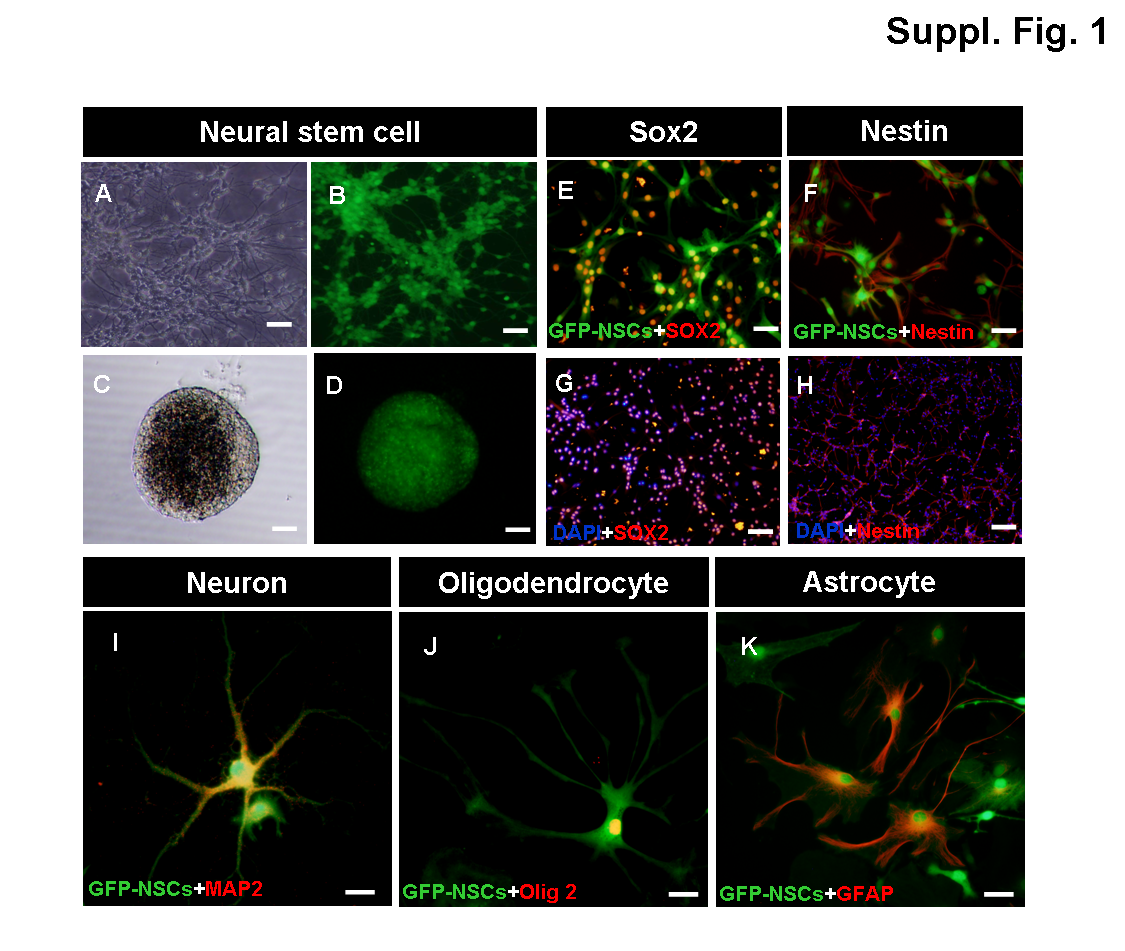


**Fig. S1. Culture of Expandable Neural Stem Cells from GFP Expressing Transgenic Mice.** (A -D) The expandable neural stem cells (NSCs) can be cultured as an adherent type (A and B) or neurospheres (C and D) with significant GFP expression. (E and F) ICC staining of Sox2 and Nestin. (G and H) Cultured NSCs are highly immunoreactive to Sox2 and Nestin. (I-K) ICC of MAP2, Olig2, and GFAP indicates that NSCs can differentiate into neurons, oligodendrocytes and astrrocytes after induction of neural differentiation. Scale bar A -D, 50 μm; E and F, 25 μm; G and H, 50 μm; I–K,10 μm.


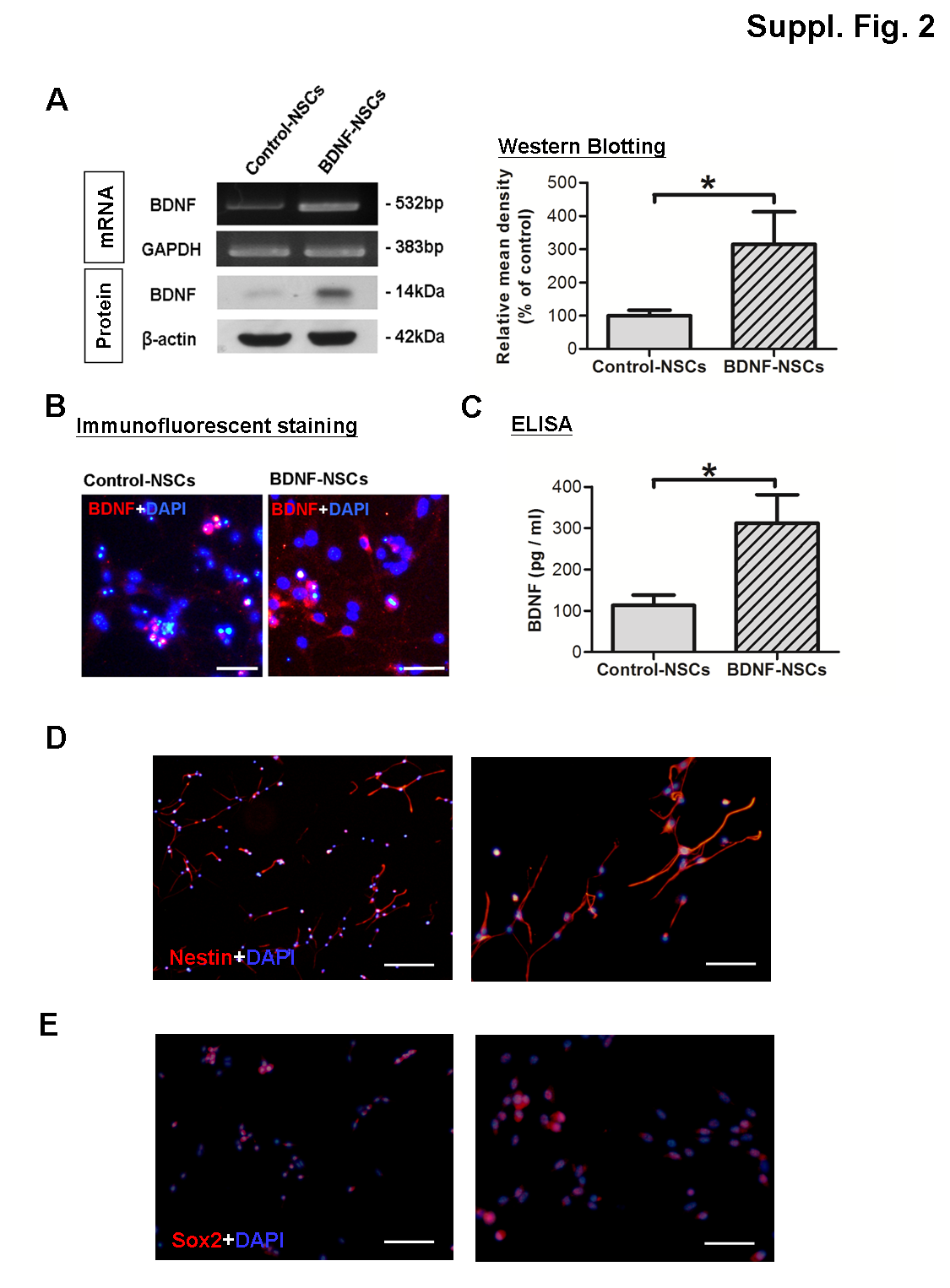


**Fig. S2. BDNF Level and the Stem Cell Markers of BDNF Overexpressing NSCs.** (A) Representative data of BDNF PCR and western blot at 48 h after electroporation. Quantification of the western blot indicates the level of overexpressed BDNF in BDNF-NSCs. Results represent mean ± SEM of 3 independent experiments. ******p* < 0.05 by unpaired *t*-test. (B) IF staining of BDNF shows BDNF-NSCs expressing higher levels of BDNF than Control-NSCs. Bar, 50 μm. (C) BDNF ELISA indicating the BDNF level is significantly higher in BDNF-NSCs. Results represent mean ± SEM of 6 independent experiments. ******p* < 0.05 by unpaired *t*-test. (D and E) IF staining of Nestin and Sox2 shows the multipotency and self-renewing properties of NSCs before transplantation. Bar, 100 μm (left), 200 μm (right).


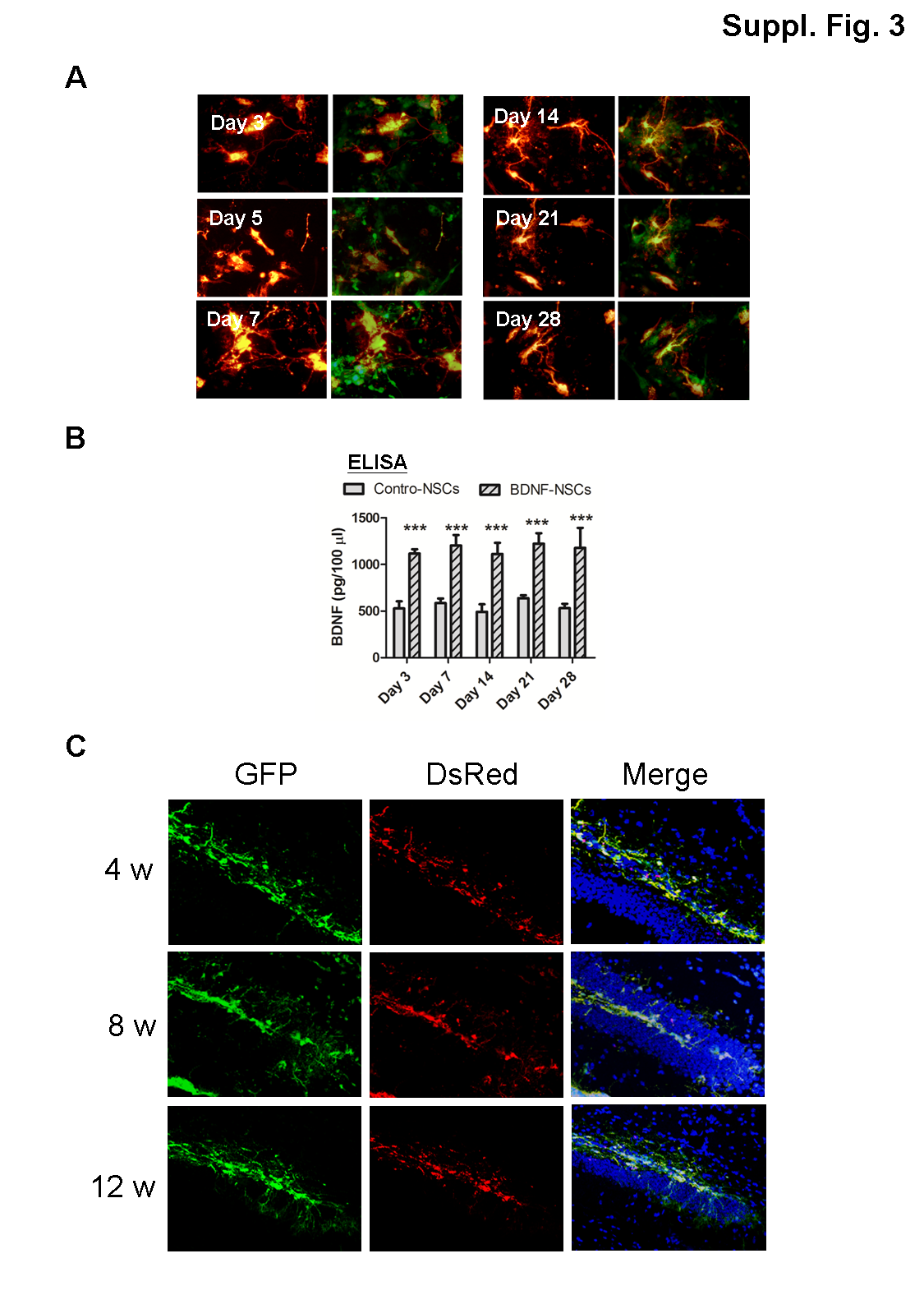


**Fig. S3. Electroporation Achieves a Stable Expression of BDNF.** (A) To test the expressional efficiency and time of BDNF in the selected cells, we additionally electroporated the pFUGW construct containing a DsRed-BDNF fragment into the cultured NSCs and evaluated the DsRed expression over the time course. The DsRed expression is highly efficient and stable in the days after electroporation. (B) ELISA of the secreted BDNF level *in vitro* from electroporated cells over the time course. Results represent mean ± SEM of 5 independent experiments. ********p* < 0.001 by unpaired *t*-test. (C) The DsRed expression from engrafted cells *in vivo*. IF staining of GFP and DsRed indicates engrafted cells stably expressing DsRed after transplantation for 4, 8, 12 weeks in the dentate gyrus of AD mice.


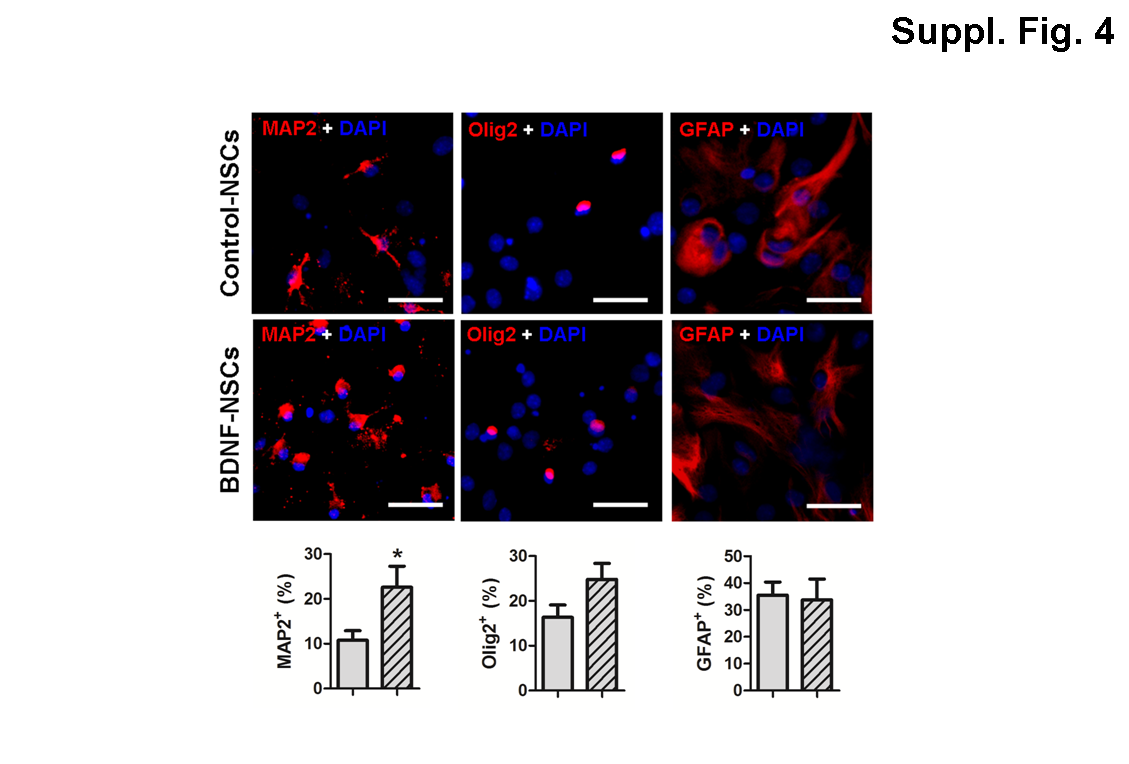


**Fig. S4. BDNF Gene Transfer Improves the Neuronal Fate of NSCs.** To detect if BDNF gene transfer altered the fate of NSCs, we induced the NSCs to differentiate into neural cells and evaluated the differentiation ratio by IF staining of MAP2, Olig2, and GFAP. The data show that BDNF gene transfer promoted NSCs to differentiate into neurons but not astrocytes or oligodendrocytes Bar, 50 μm. Results represent mean ± SEM of 6 independent experiments. ******p* < 0.05 by unpaired *t*-test.


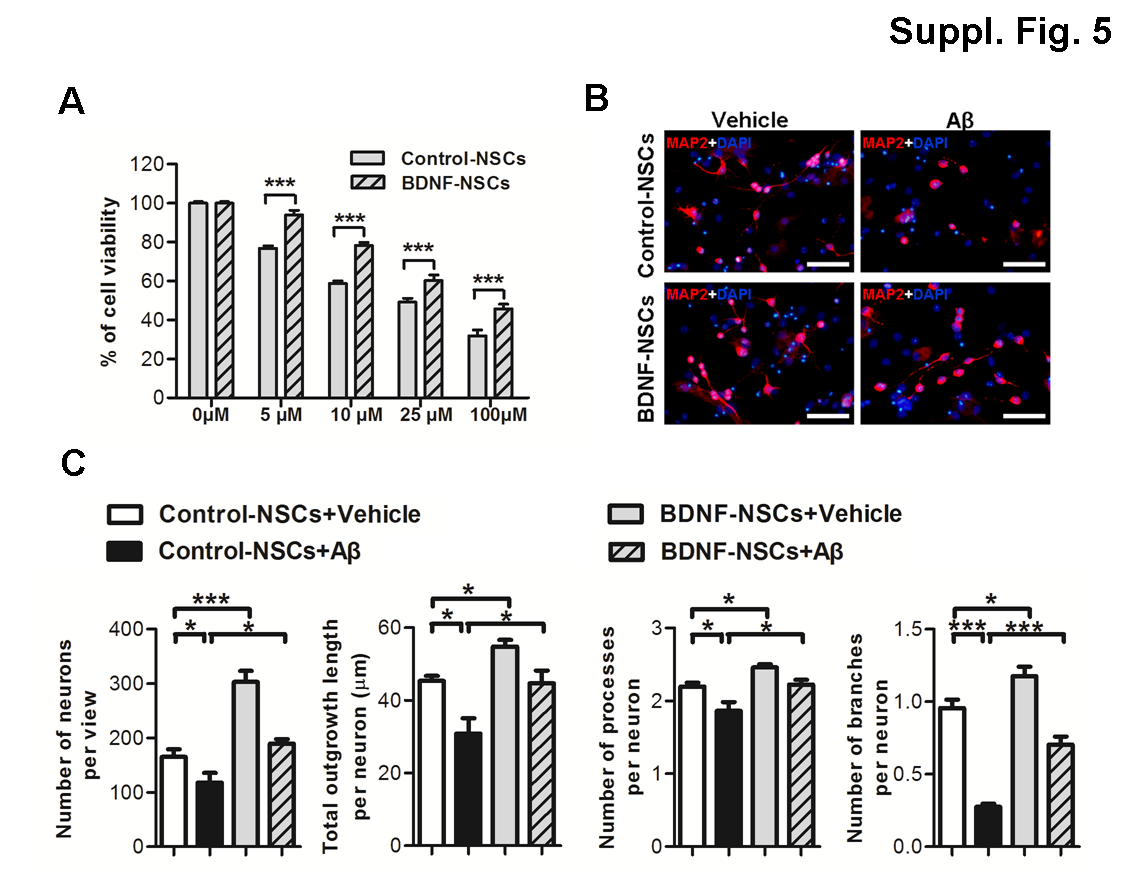


**Figure S5. BDNF Overexpression Maintains the Cell Viability, Neuronal Fate, and Neurite Outgrowth of NSCs in Aβ Toxicity.** (A) MTT assay of cultured NSCs in a gradient of Aβ_42_ concentrations. Results represent the mean ± SEM of six independent experiments. ********p* < 0.001 by two-way repeated measure ANOVAs with Bonferroni multiple comparisons tests. (B) IF staining of MAP2 (red) for the Control-NSC- and BDNF-NSC-derived neurons in vehicle or 10 μM of Aβ_42_. Scale bar: 50 μm. (C) High-throughput screen analysis of IF staining for MAP2 indicates the number of differentiated neurons, the total length of neurite outgrowth, and the number of processes and branches. Results are presented as the mean ± SEM of eight independent experiments. ******p* < 0.05; *******p* < 0.01; ********p* < 0.001 by one-way ANOVAs with Tukey’s post hoc tests


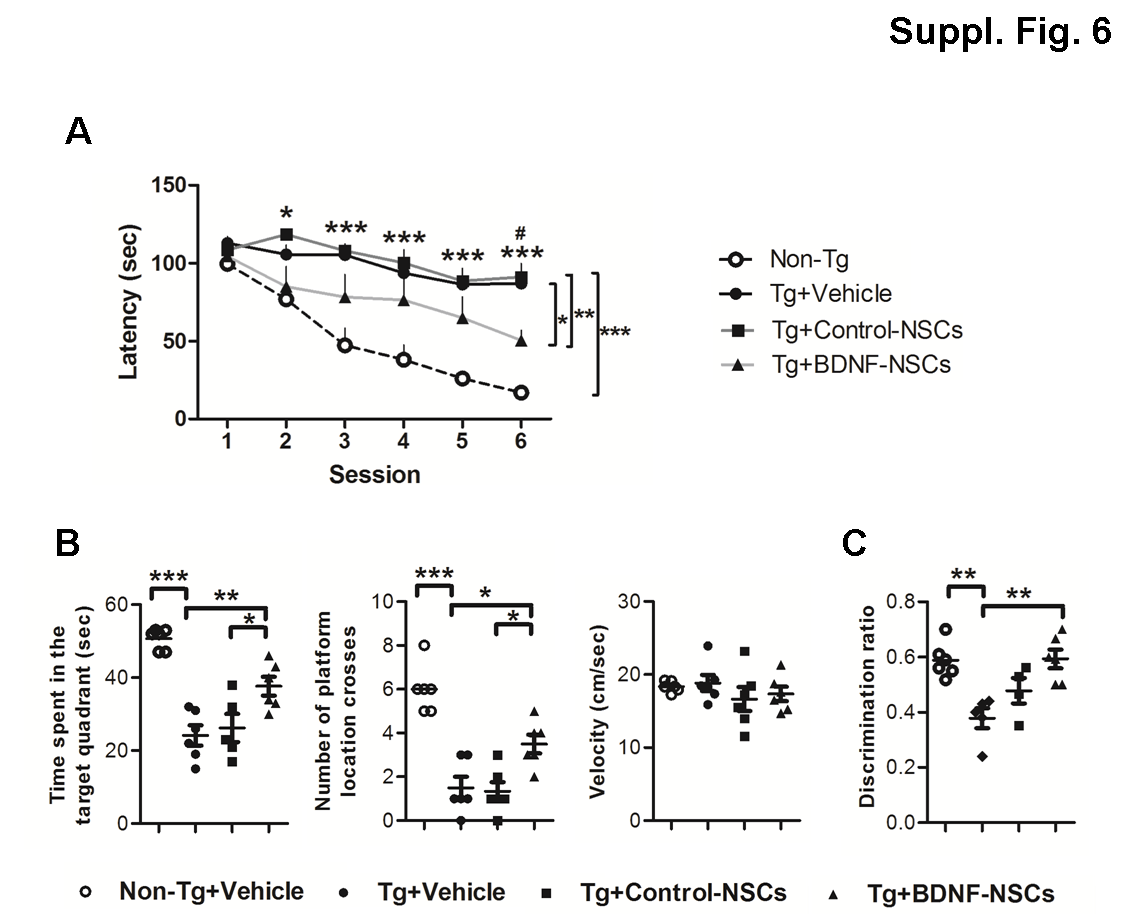


**Figure S6. Transplantation of BDNF-NSCs reveals longterm therapeutic effects at 3-mon after transplantation.** (A) The escape latency to find the hidden platform in the MWM task (n = 6 per group; *Non-Tg mice vs. Tg+Vehicle mice, ^#^ Tg+Vehicle mice vs. Tg+BDNF-NSCs mice). The multiple comparisons test confirmed the significant differences between each group. (B) The records of the search time in the target quadrant, the number of crosses of the probe location, and swimming velocity in the probe trial test (n = 6 per group). (C) The discrimination ratio of NOR test (discrimination index= novel object exploration time / total exploration time) (n = 6 per group). All results are presented as the mean ± SEM. * or one symbol represent *p* < 0.05, ** *p* < 0.01, *** *p* < 0.001. The data of escape latency and novel object recognition were analyzed by two-way ANOVAs with Bonferroni post-tests, the others were analyzed by one-way ANOVAs with Tukey’s post hoc tests.


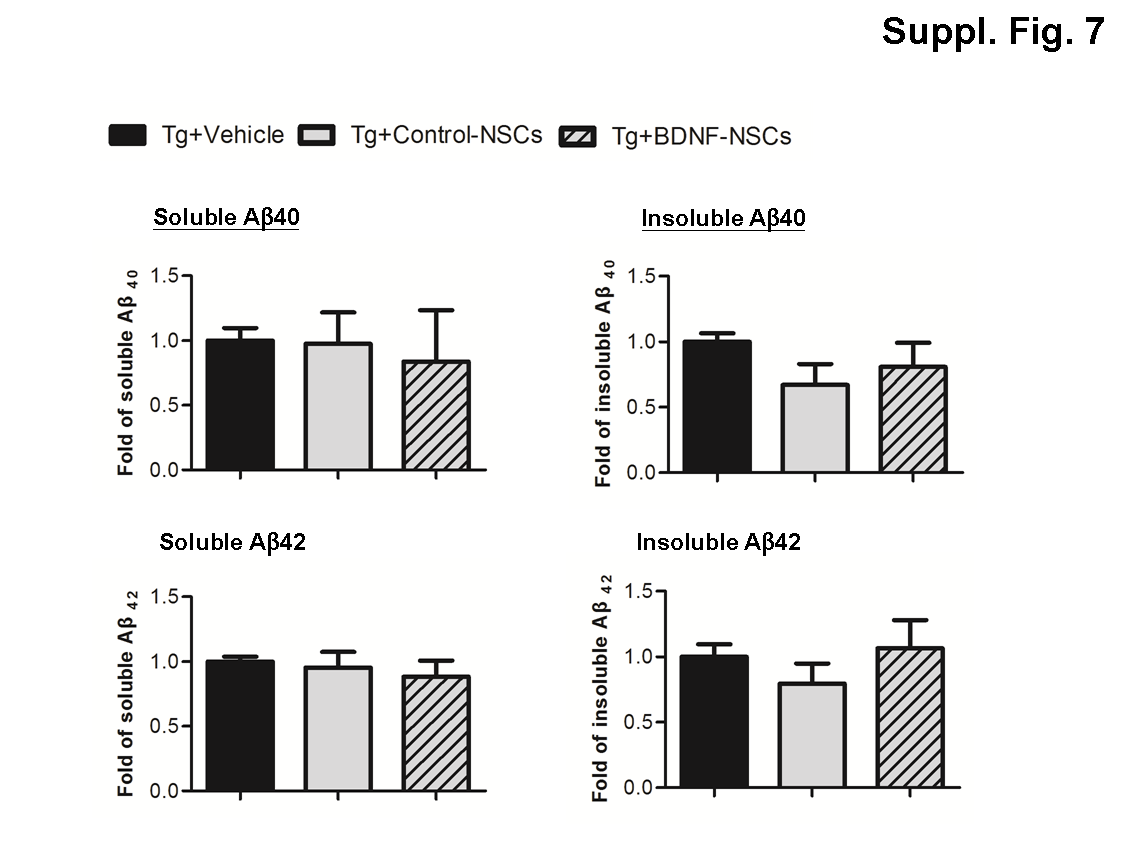


**Fig. S7. ELISA of Soluble and Insoluble Aβ40 and Aβ42.** To determine whether engrafted NSCs took part in Aβ clearance, we determined the concentration of soluble and insoluble Aβ40 and Aβ42 in AD mice at 4 weeks after NSCs transplantation. The data show that the hippocampal soluble and insoluble Aβ40 and Aβ42 level are no different in each group (n=5 per group).


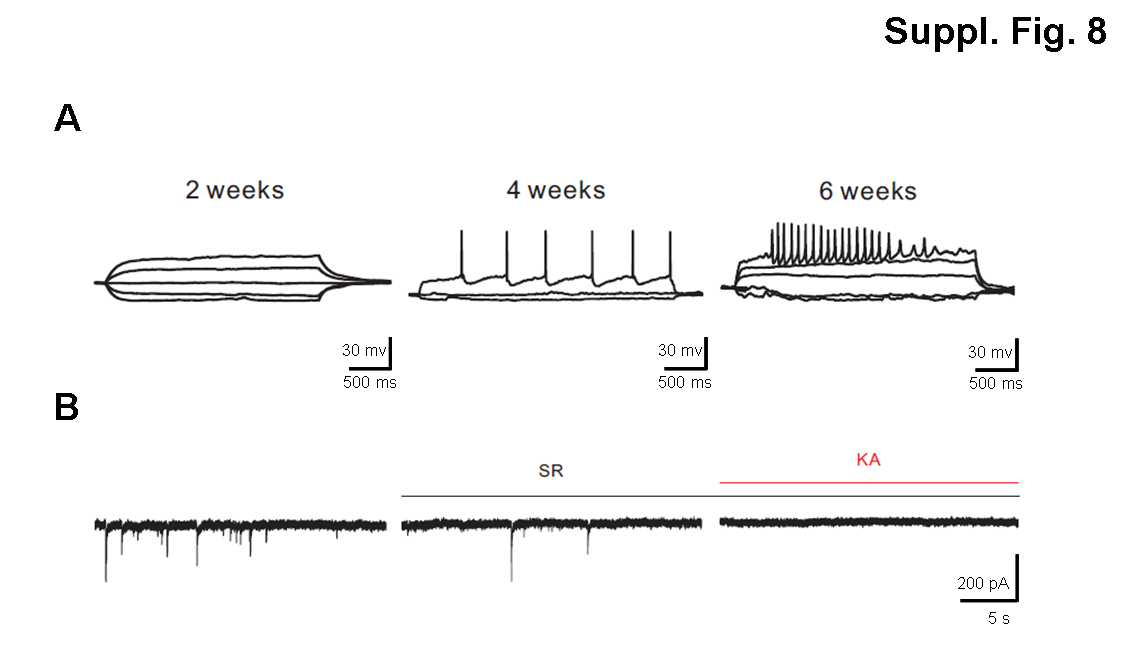


**Fig. S8. Engrafted Cell-derived Neurons Display Electrophysiological Properties of Mature Neuron 4 Weeks After Transplantation Into Non-tg mice.** (A) Representative figures of AP firing pattern from engrafted cell-derived neurons at 2, 4, and 6 weeks after transplantation. Data represent by BDNF-NSC. (B) Spontaneous responses recorded from engrafted cell-derived neuron. The spontaneous events were blocked by the addition of AMPA/NMDA (KA) and GABAergic receptor blocker (SR). Data represent by BDNF-NSC. (Control-NSCs n = 5 from 3 mice, BDNF-NSCs n = 5 from 3 mice).

Table S1. Pearson Correlation Analysis of Behavioral Outcomes with the Number of Surviving NSCs, Number and Ratio of Surviving NSC-derived Neurons, and BDNF level

|  | Mean number of surviving NSCs | Mean number of NSC-derived neurons | Mean ratio of NSC-derived neurons | BDNF level |
| --- | --- | --- | --- | --- |
| **Latency at Session 6** | *r* = -0.8522  *p* = 0.0004******* | *r* = -0.6590  *p* = 0.0198***** | *r* = -0.6371  *p* = 0.0276***** | *r* = -0.8438  *p* = 0.0006******* |
| **Number of platform  location crosses** | *r* = 0.7033  *p* = 0.0107***** | *r* = 0.7442  *p* = 0.0055****** | *r* = 0.7403  *p* = 0.0056****** | *r* = 0.6716  *p* = 0.0168***** |

******p* < 0.05; *******p* < 0.01; ********p* < 0.001.

**Table S2. Comparison between the Studies by Blurton-Jones et al. and Wu et al.**

|  | | **Blurton-Jones et al.** | **Wu et al.** | Note |
| --- | --- | --- | --- | --- |
| Key axis | | BDNF trophic effects on AD-Tg mice | BDNF trophic effects on AD-Tg mice and engrafted cells |  |
| The effects of BDNF on engrafted cells | Viability | N/A | The viability of engrafted cells declines after transplantation into the AD brain. BDNF-NSCs are better able to survive than Control-NSCs. | A statistical correlation between engrafted cell viability and cognitive amelioration of AD-Tg mice. |
|  | Neuronal fate ratio | N/A | Both kinds of engrafted cells can differentiate into neurons after transplantation. BDNF overexpression improves the neuronal fate ratio of engrafted cells. | A statistical correlation between neuronal fate ratio of engrafted cells and cognitive amelioration of AD-Tg mice. |
|  | Electrophysiological properties | N/A | At 4 weeks after transplantation, both kinds of cells can generate action potentials (APs) in the brain of age matched non-Tg mice; however, they generate APs until 8 weeks after transplantation in the AD brain. BDNF-NSC-derived neurons exhibit a lower input resistance, higher capacitance, higher AP firing ratio, lower AP threshold, and higher AP amplitude, suggesting BDNF overexpression improves the neuronal maturation of excitability in engrafted cells. |  |
|  | Ability of functional network integration in host brain | N/A | Both kinds of engrafted cells integrate into Ca^2+^-mediated functional fluctuation networks at 4 weeks after transplantation and hippocampal GABA and glutamatergic synaptic circuits of AD mice at 8 weeks after transplantation. |  |
|  | Neurite complexity of engrafted cells | N/A | BDNF overexpression improves the neurite outgrowth of engrafted cells. |  |
|  | Synaptic density of engrafted cells | N/A | BDNF overexpression improves the functional neuronal maturation in the development of synaptic spine density and excitatory synapses with AMPAR. |  |
| The effects of BDNF on AD mice | Aβ level | Aβ level is not altered | Aβ level is not altered | Soluble and insoluble Aβ levels were determined by Blurton-Jones et al. at 4 weeks and Wu et al. at 8 weeks after NSC transplantation. |
|  | BDNF level | NSC transplantation recovers the decreased BDNF levels of AD-Tg mice. | Both Control-NSC and BDNF-NSC transplantation recover BDNF levels of AD-Tg mice, but BDNF-NSC transplantation reveals a better efficacy. | A statistical correlation between BDNF level and cognitive amelioration of AD-Tg mice in the study by Wu et al. |
|  | Synaptic density | NSC transplantation recovers the synaptic density of AD-Tg mice by BDNF trophic effects; the synaptic recovery is also achieved by direct hippocampal injection of recombinant BDNF. | Both Control-NSC and BDNF-NSC transplantation recover the synaptic density of AD-Tg mice, but BDNF-NSC transplantation reveals a better efficacy. In addition to BDNF trophic effects, engrafted BDNF-NSCs display better ability of synaptic development to contribute to the AD brain. | Wu et al. found that BDNF-NSCs are better able to survive, differentiate into neurons, and behave as functionally matured neurons after transplantation. Thus, BDNF-NSC transplanted AD mice exhibit better recovery of synaptic density. |
|  | Behaviors | NSC transplantation ameliorates the cognitive deficits of AD-Tg mice by BDNF trophic effects. Similar beneficial effects are achieved by direct hippocampal injection of recombinant BDNF. | Both Control-NSC and BDNF-NSC transplantation improve the cognitive deficits of AD-Tg mice, BDNF-NSC reveals better therapeutic efficacy not only through trophic effects but also through functional cell replacement. | Blurton-Jones et al examined the behavioral outcomes 4 weeks after NSC transplantation without touching the events of NSC-based neuronal replacement or circuit reconstruction. Wu et al. examined that 8 weeks after NSC transplantation and found the feasibility of engrafted cells acting as functional neurons contributing to the AD brain. |
|  | Long-term effects | N/A | Three months after transplantation, Control-NSC lost the therapeutic efficacy for cognitive behaviors of AD-Tg mice, but BDNF-NSC still exhibited the therapeutic potential. | Three months in aged mice is equivalent to approximately 6 years in humans [14]. |
| Conclusion | | NSC transplantation improves the AD cognitive deficits through BDNF trophic effects. | Gain of BDNF function in engrafted cells improves its therapeutic potential not only through a better trophic effect, but also through functional neuronal replacement. |  |
| Implication for AD therapy | | The BDNF trophic effects of naive NSC transplantation may be achieved by direct intra-hippocampal injection. Thus, approaches for the increase of brain BDNF levels may be developed as strategies. | In addition to BDNF trophic effects, engrafted cells can behave as functional neurons and integrate into endogenous hippocampal circuits for recovery of the AD cognitive deficits. Appropriate cell resources for autologous transplant combined with BDNF overexpression may be a promising strategy. |  |

N/A: non-applicable

**SUPPLEMENTARY LEGENDS FOR THE VIDEOS**

**Suppl. Video 1. In Vivo Confocal Endoscope-based Video of Engrafted BDNF-NSCs in the Living Mouse Brain.** Representative video of engrafted BDNF-NSCs showing the engrafted NSCs survived and migrated in the hippocampus of AD-Tg mice 4 weeks after transplantation. The quantification of GFP-expressing cells from Control-NSCs and BDNF-NSCs by relative fluorescence units is shown in Fig. 2b.

**Suppl. Video 2. Ex Vivo Calcium Imaging of Engrafted BDNF-NSC.** Representative video of calcium imaging from the engrafted BDNF-NSC showing the application of high potassium chloride elicited responses of calcium fluctuation from engrafted and endogenous cells. Images of radiometric calcium orange [Ca^2+^]_i_ indicated the engrafted cells were able to integrate into Ca^2+^-mediated functional networks, the Ca^2+^-waves propagated within engrafted cells and neighboring endogenous cells.

**SUPPLEMENTARY MATERIAL AND METHODS**

**Primary Culture of GFP-expressing NSCs and the Induction of Differentiation**

GFP-expressing NSCs or neural progenitors were derived from a transgenic mouse line with “enhanced” GFP cDNA (Jackson Labs). The GFP-expressing NSCs were harvested from the hippocampi of postnatal day 1 pups and expanded in adherent type, as described previously [1]. Each batch of NSCs was cultured for 10 days *in vitro*, and then conducted to BDNF or control vector electroporation. NSCs for the MTT and neurite outgrowth assays were prepared from adult C57Bl/6J mice (purchased from the animal center of NCKU). For the induction of spontaneous neural cell differentiation, NSCs were plated on poly-L-ornithine (Sigma, P3655) and laminin-coated (BD, 354232) culture dishes and then replaced with mitogen-free medium for 7 days [2].

**Western blotting, ELISAs, and RT-PCR**

To analyze the BDNF level, the cell extracts were prepared 48 h after construction transfection *in vitro* by homogenization of the cells in radio-immunoprecipitation assay lysis buffer. To analyze the hippocampal BDNF level in AD mice, extracts were prepared from the hippocampus of non-Tg or AD mice. The detailed protocol for western blotting has been described previously [3]. The extracts were analyzed by 8–12% sodium dodecyl sulfate polyacrylamide gel electrophoresis, followed by blot hybridization with the following anti-bodies: BDNF (1:1000; Genetex, GTX62495), SYP (1:5000; Millipore, MAB5658), PSD95 (1:2000; Millipore, HAPN68), α-tubulin (1:10000; Millipore, 05-829), and β-actin (1:10000; Millipore, MAB1501). Secondary antibodies were horseradish peroxidase-linked (1:50000; PerkinElmer, NEF81200IEA or NEF82200IEA).

Image J software was used to quantify the relative intensities of the bands, which were normalized to the intensity of actin or tubulin. The results are presented as the mean ± SEM. The BDNF ELISA was performed following the manufacturer’s protocol (Promega). For BDNF PCR analysis, the following primers were used:

5ʹ-CAGTGGACATGTCTGGCGGGACGGTC-3ʹ and 3ʹ-TTCTTGGCAACGGCAACAAACCACAAC-5ʹ.

For GAPDH (glyceraldehyde-3-phosphate dehydrogenase), 5ʹ-GACCCCTTCATTGACCTCAAC-3ʹ and 3ʹ-TCTTACTCCTTGGAGGCCATG-5ʹ were used.

**MTT Assay**

To test the viability of NSCs in Aβ toxicity, cultured NSCs were plated onto poly-L-ornithine-coated and laminin-coated 96-well plates at 48 h following construction transfection. The dose-dependent responses were tested using a gradient of Aβ_42_ concentrations (ANASPECT, 20276) from 5 μM to 100 μM, preparation as previously described [4]. The MTT assay for cell viability followed the previous study [5].

**Neurite Outgrowth Assay**

To test the performance of neurite outgrowth during NSC differentiation in Aβ toxicity, NSCs were plated onto 96-well coated plates. After inducing differentiation for 7 days in 5 μM Aβ_42_, the cells were fixed with 4% paraformaldehyde and immunocytochemistry was performed for MAP2.

To quantify the neurite outgrowth, immuno-MAP2 and DAPI double-positive cells were acquired by the ImageXpress System (Molecular Devices). The Neurite Outgrowth Module of the MetaXpress software (Molecular Devices) was used to analyze the number of neurons, length of total outgrowth per cell, and number of processes and branches per cell.

**Nest Construction Test**

All of behavioral tests were performed in blind manner. The nest construction test was conducted as described in [6]. Briefly, starting at 17:00, individual cages were supplied with a 5 cm^2^ piece of paper towel. The next morning (09:00), the nest in each cage was photographed and scored in a 3-point manner: 1, no biting or tears on the paper; 2, moderate biting and/or tears on the paper, but no consistent nest observed in the cage corners; and 3, most of the paper was torn into approximately 1-cm pieces and grouped into a corner of the cage.

**Morris Water Maze Task**

For testing the spatial learning/memory of the mice, the MWM task was performed at 8 weeks after NSC transplantation as described previously [4]. All experiments were performed in a blinded manner. The animals were subjected to one session per day, which consisted of four trials. For a complete test, six sessions were administered. The time that it took the mouse in each trial to reach the platform in the water was recorded as the escape latency. After 6 days of escape training, the platform was removed for the probe trial test, which tested the retention of spatial memory at 24 h after training. The number of crossing the probe and the time spent in the target quadrant were recorded by the video and quantified in blind manner.

**Novel Object Recognition Test**

For the novel object recognition test at 8 weeks after NSC transplantation, the mice were replaced in a new open-field cage (35 × 45 × 40 cm), as per the methods and rules followed in previous studies [7, 8]. On training day 1, the mice were placed individually in the cage for a 5 min familiarization trial with no objects. Subsequently, the mice went through three sessions of 5 min sample trials with two of the same objects positioned at specific places in the cage. Each trial was separated by a 3 min interval period where the mice were returned to their original cages. Twenty-four hours later, the mice were subjected to the test trial for 5 min with two objects in the cage (a familiar object and a novel object) positioned at the same specific places. The discrimination index (DI) was calculated as follows: DI = novel object exploration time/total exploration time.

**Immunocytochenistry Staining**

Immunocytochemiostry (ICC) staining was used to examine the expression of BDNF, multi-potency of NSCs, and neurite outgrowth of NSC-derived neurons. For ICC staining, following fixation with 4% PFA and blocking in 5% goat serum and 0.01% Triton-X 100 in PBS for 1 h at room temperature, cells were incubated overnight at 4°C in blocking buffer containing primary antibodies that recognize BDNF (1:300; Genetex, GTX62495), MAP2 (1:200; Millipore AB5622), GFAP (1:500; Millipore, MAB3402), and Olig2 (1:300; Millipore, AB9610). A series of Alexa Fluor-conjugated secondary antibodies (1:300; Invitrogen) were used to recognize the primary antibodies. The ICC staining data were photographed by an immunofluorescent microscope (Olympus, IX71) or high-throughput screening microscope (Molecular Devices, ImageXpress).

**Immunofluorescence Staining**

To prepare the tissue for immunofluorescence (IF) staining, adult mice were anesthetized and perfused transcardially by PBS and 4% paraformaldehyde. The brain of each mouse was removed and immersed in a 4% PFA solution for 2 h and dehydrated by gradients of 15%, 20%, and 30% sucrose. After preparing the cryosections, the tissues were incubated with a series of primary antibodies: MAP2, GFAP (same as immunocytochemical staining), DCX (1:300; Millipore, AB5910), NeuN (1:300; Millipore, MAB377), GFP antibody (Millipore, MAB3080 or 3580), SYP (1:500; Millipore, MAB5658), GluR2 (1:200; Abcam, ab133477; Alexa Fluor-conjugated secondary antibodies (1:300; Invitrogen) were used to recognize the primary antibodies. The sections were then incubated with DAPI and coverslipped with the mounting medium (Dako). All sections were examined by a laser scanning confocal microscope (Nikon C1-Si) or acquired by TissueFAXS Plus Cytometry (TissueGnostics).

**Data Quantification**

Quantification of the differentiated neural cells *in vitro* followed the methods that previous described [9]. The differentiated neural cells were counted in a blinded manner, and around 2000 cells were quantified from 10 randomly selected fields in each group. The DAPI-labeled cells were quantified as total number of cells, and the proportions of cells that were double labeled with MAP2, GFAP, and Olig2 were calculated.

For quantifying the survival of GFP-expressing cells and GFP-NSC-derived neurons, whole stereologic images were acquired by TissueFAXS and analyzed by TissueQuest (TissueGnostics) as previously described [10]. Stereologic evaluations of the hippocampus of each experimental mouse were conducted on every 12^th^ section throughout the hippocampus that contained GFP signals following the Cavalieri principle [9]. Flow cytometry-like data (dot-plot) were acquired by TissueQuest, with each dot of the plot representing two specific kinds of immunoreactive intensity from a single cell. The plot accumulated the values of all cells from a region of interest, and then the positive signal number could be identified by gating the immunoreactive intensity.

Quantification of the PSD95 density was performed following methods that were modified from a previous study [11]. Z-stack images (40 slices, 0.3 μm per step size) were acquired using a consistent setting in each batch of samples. Subsequently, 30–40 square regions of interest that were randomly selected within the inner molecular layer of dentate gyrus were converted to grayscale images. We then analyzed the mean pixel intensity of these images using Image J. The background intensity measured within the lateral ventricle was subtracted from the mean pixel intensity. Quantification of GluR2 and SYP overlay number, Z-stack projected images were acquired using a consistent setting in each batch of samples. GFP expressing cells integrated into the DG were identified and counted the number of colocalized immunoreactivity of GluR2 and SYP within the dendrites of each neuron. 7-8 neurons in the DG were randomly selected per slide from a mouse, total 3 mice were analyzed per group.

**Slice Preparation and Electrophysiological Recording**

Mice were killed by rapid decapitation in accordance with the guidelines of animal center of National Yang-Ming University. Their brains were rapidly removed, and 300 μm-thick coronal hippocampal slices were cut in ice-cold sucrose solution containing the following (in mM): 87 NaCl, 25 NaHCO_3_, 1.25 NaH_2_PO_4_, 2.5 KCl, 10 glucose, 75 sucrose, 0.5 CaCl_2_, and 7 MgCl_2_ using a vibratome (DTK-1000, Dosaka). Slices were incubated in the sucrose solution (equilibrated with 95% O_2_ and 5% CO_2_) in a holding chamber at 34°C for 30 min and kept in the same chamber at room temperature (23 ± 2°C) until used. During the experiments, slices were placed in a recording chamber and superfused with oxygenated artificial CSF (ACSF) containing the following (in mM): 125 NaCl, 25 NaHCO_3_, 1.25 NaH_2_PO_4_, 2.5 KCl, 25 glucose, 2 CaCl_2_, and 1 MgCl_2_. The recording temperature was 23 ± 2°C. Recording electrodes (3–7 MΩ) were pulled from borosilicate glass (outer diameter, 1.5 mm; thickness, 0.32 mm; Harvard apparatus). GFP^+^ cells were first identified by the fluorescence with the soma located in the inner molecular layer, granule cell layer, or the hilus under an infrared and differential interference contrast microscope (Olympus BX51WI) coupled with an infrared-sensitive CCD camera (Hamamatsu, C7500–50). Recorded cells (held at -70 mV in current clamp) were depolarized to generate APs by somatic current pulse injection.

In the experiments where spontaneous EPSCs and IPSCs were recorded, cells were held at -55 mV. When recording evoked glutamatergic/GABAergic currents, a stimulation electrode was placed in the inner molecular layer. Single short (0.1 ms duration) current pulses were delivered to evoke synaptic responses. Cells were held at -75 or 0 mV. Whole-cell patch-clamp recordings were made using a Multiclamp 700B amplifier (Molecular Devices). Pipette capacitances of both electrodes were carefully compensated (by ~95%), and series resistance (Rs) was compensated using the automatic bridge balance (readouts after compensation were 9–21 MΩ). Signals were filtered at 4 kHz using the 4-pole low-pass Bessel filter. A Digidata 1440A (Molecular Devices) connected to a personal computer was used for stimulus generation and data acquisition. The sampling frequency was 10 kHz. Pulse sequences were generated by pClamp 10.2 (Molecular Devices).

For the majority of the whole-cell recordings, the intracellular solution contained the following (mM): 136.8 K-gluconate, 7.2 KCl, 0.2 EGTA, 4 MgATP, 10 HEPES, 7 Na_2_-phosphocreatine, and 0.4% biocytin. In some experiments, intracellular solutions with high Cl^-^ (144 mM) concentrations were used to amplify the amplitude of GABAergic events. High Cl^-^-containing intracellular solutions contained (mM): 144 KCl, 0.2 EGTA, 4 MgATP, 10 HEPES, 7 Na2-phosphocreatine, 0.1 GTP, and 0.4% biocytin, pH adjusted to 7.3 with KOH. One or more of the following antagonists were also included in the ACSF: 2 mM kynurenic acid (Sigma) to block AMPA and NMDA receptors and/or 1 μM gabazine (Tocris) to block GABA_A_ receptors.

Data were analyzed using Clampfit 10.3 (Molecular Devices) and Prism 5.0 (GraphPad). The input resistance (R_in_) was measured by the ratio of the steady-state (the last 100 ms) voltage response versus the injected 2-s hyperpolarizing (10 pA) current pulse. The spike threshold was measured as the voltage at which the first derivative of voltage exceeded the threshold (20 V/s; see Campanac et al, 2013). [12]

**Sholl Analysis**

Sholl analysis [13] was used to investigate dendritic complexity in relation to their distance from the soma. Briefly, the reconstructed neuronal morphology was conducted by biocytin-filling during cell recording and the concentric Sholl segments (concentric radial interval: 10 μm) were generated starting at a distance of 10 μm from the center of the soma and the number of process intersections was analyzed per Sholl segment, respectively.

**REFERENCES**

1. Gage, FH, Kempermann, G, Palmer, TD, Peterson, DA, and Ray, J (1998). Multipotent progenitor cells in the adult dentate gyrus. *J Neurobiol* **36**: 249-266.

2. Brannvall, K, Bergman, K, Wallenquist, U, Svahn, S, Bowden, T, Hilborn, J*, et al.* (2007). Enhanced neuronal differentiation in a three-dimensional collagen-hyaluronan matrix. *J Neurosci Res* **85**: 2138-2146.

3. Tsai, KJ, Yang, CH, Fang, YH, Cho, KH, Chien, WL, Wang, WT*, et al.* (2010). Elevated expression of TDP-43 in the forebrain of mice is sufficient to cause neurological and pathological phenotypes mimicking FTLD-U. *J Exp Med* **207**: 1661-1673.

4. Tsai, KJ, Tsai, YC, and Shen, CK (2007). G-CSF rescues the memory impairment of animal models of Alzheimer's disease. *J Exp Med* **204**: 1273-1280.

5. Eucher, JN, Uemura, E, Sakaguchi, DS, and Greenlee, MHW (2007). Amyloid-beta peptide affects viability but not differentiation of embryonic and adult rat hippocampal progenitor cells. *Experimental Neurology* **203**: 486-492.

6. Wesson, DW, and Wilson, DA (2011). Age and gene overexpression interact to abolish nesting behavior in Tg2576 amyloid precursor protein (APP) mice. *Behav Brain Res* **216**: 408-413.

7. Grillo, FW, Song, S, Teles-Grilo Ruivo, LM, Huang, L, Gao, G, Knott, GW*, et al.* (2013). Increased axonal bouton dynamics in the aging mouse cortex. *Proc Natl Acad Sci U S A* **110**: E1514-1523.

8. Mansuy, IM, Mayford, M, Jacob, B, Kandel, ER, and Bach, ME (1998). Restricted and regulated overexpression reveals calcineurin as a key component in the transition from short-term to long-term memory. *Cell* **92**: 39-49.

9. Blurton-Jones, M, Kitazawa, M, Martinez-Coria, H, Castello, NA, Muller, FJ, Loring, JF*, et al.* (2009). Neural stem cells improve cognition via BDNF in a transgenic model of Alzheimer disease. *Proc Natl Acad Sci U S A* **106**: 13594-13599.

10. Wang, IF, Guo, BS, Liu, YC, Wu, CC, Yang, CH, Tsai, KJ*, et al.* (2012). Autophagy activators rescue and alleviate pathogenesis of a mouse model with proteinopathies of the TAR DNA-binding protein 43. *Proc Natl Acad Sci U S A* **109**: 15024-15029.

11. Graff, J, Rei, D, Guan, JS, Wang, WY, Seo, J, Hennig, KM*, et al.* (2012). An epigenetic blockade of cognitive functions in the neurodegenerating brain. *Nature* **483**: 222-226.

12. Campanac, E, Gasselin, C, Baude, A, Rama, S, Ankri, N, and Debanne, D (2013). Enhanced intrinsic excitability in basket cells maintains excitatory-inhibitory balance in hippocampal circuits. *Neuron* **77**: 712-722.

13. Sholl, DA (1953). Dendritic organization in the neurons of the visual and motor cortices of the cat. *J Anat* **87**: 387-406.

14. Flurkey ,K, Currer, JM, and Harrison, DE, (2007). The Mouse in Aging Research. In The Mouse in Biomedical Research 2nd Edition. Fox JG, et al, editors. American College Laboratory Animal Medicine (Elsevier), Burlington, MA. pp. 637–672.).
